# Maximizing the accuracy of genetic variance estimation and using a novel generalized effective sample size to improve simulations

**DOI:** 10.1007/s00122-025-04861-8

**Published:** 2025-03-18

**Authors:** Javier Fernández-González, Julio Isidro y Sánchez

**Affiliations:** Centro de Biotecnologia y Genómica de Plantas (CBGP, UPM-INIA), Universidad Politécnica de Madrid (UPM) - Instituto Nacional de Investigación y Tecnologia Agraria y Alimentaria (INIA), Campus de Montegancedo-UPM, 28223 Pozuelo de Alarcón, Madrid Spain

## Abstract

**Key message:**

We developed an improved variance estimation that incorporates prediction error variance as a correction factor, alongside a novel generalized effective sample size to enhance simulations. This approach enables precise control of variance components, accommodating for more flexible and accurate simulations.

**Abstract:**

Phenotypic variation in field trials results from genetic and environmental factors, and understanding this variation is critical for breeding program simulations. Additive genetic variance, a key component, is often estimated using linear mixed models (LMM), but can be biased due to improper scaling of the genomic relationship matrix. Here, we show that this bias can be minimized by incorporating prediction error variance (PEV) as a correction factor. Our results demonstrate that the PEV-based estimation of additive variance significantly improves accuracy, with root mean square errors orders of magnitude lower than traditional methods. This improved accuracy enables more realistic simulations, and we introduce a novel generalized effective sample size (ESS) to further refine simulations by accounting for sampling variation. Our method outperforms standard simulation approaches, allowing flexibility to include complex interactions such as genotype by environment effects. These findings provide a robust framework for variance estimation and simulation in genetic studies, with broad applicability to breeding programs.

**Supplementary Information:**

The online version contains supplementary material available at 10.1007/s00122-025-04861-8.

## Introduction

Phenotypic variation in field trials arises from numerous components, including additive and non-additive genetic effects, environmental effects, spatial variation within the field, and measurement errors. Partitioning of the variance involves fitting a model to the data and extracting the variance values from it. There are several available options, including linear mixed models (LMM), which are based on restricted maximum likelihood (REML); Haseman-Elston regression, based on least squares, and bayesian models, based on Gibbs sampling. Each of these methods has several advantages and disadvantages, but all of them may present bias in the estimation of variance components under certain circumstances as discussed in depth in Chen (2014, 2016), de Los Campos et al. (2015). In this work, we will focus on LMM due to their combination of computational efficiency, flexibility for including non-additive effects, good performance (Kerin and Marchini 2020) and widespread application (Hallauer et al. 2010; Coutiño-Estrada and Vidal-Martínez 2006; Carvalho et al. 2017; Yue et al. 2022; Coelho et al. 2021). The bias in the estimation can come from two main sources (de Los Campos et al. 2015): firstly, overly simplistic model assumptions that do not match the reality can bias the variance estimates. This is outside the scope of this work. Secondly, extracting a value for the variance components from a given model is not straightforward and can cause substantial additional bias. In this work, we aim to prove this issue, as well as proposing a solution, which will remove one of the main sources of bias for variance estimates from LMM.

Minimizing bias in the estimation of variance components is crucial in breeding, as they are required for the computation of heritability and the study of the genetic control of complex traits. They are also essential for estimating response to selection using the breeder’s equation (Lush 1943). The additive genetic variance $\big (\sigma ^2_a\big )$ is of special relevance as the only heritable genetic effect (Falconer and Mackay 1983) and we will focus on it for the sake of simplicity. However, our arguments apply to any other variance component as well.

Typically, in a LMM, $\sigma ^2_a$ is extracted from a random effect ${\varvec{a}} \sim \text {MVN}({\varvec{0}}, G\sigma ^2_{a^*})$, where ${\varvec{a}}$ is a vector of true breeding values, *G* is an additive genomic relationship matrix, often calculated with the VanRaden (2008) formulation, and $\sigma ^2_{a^*}$ is a scaling factor estimated by REML. It is often incorrectly assumed that $\sigma ^2_a = \sigma ^2_{a^*}$, using $\sigma ^2_{a^*}$ as the estimate for the additive variance component. This is true only if the *G* matrix is scaled to unit variance, meaning the overall variability encompassed within the multivariate covariance matrix *G* equals one. The scaling performed in the standard *G* matrix (VanRaden 2008) attempts to achieve this, but it relies on several, unrealistic assumptions outlined in Appendix 1. This results in the VanRaden matrix being improperly scaled for variance component estimation, making $\sigma ^2_{a^*}$ non-representative of the overall additive variance, i.e., $\sigma ^2_{a^*} \ne \sigma ^2_a$ (Forni et al. 2011; Lehermeier et al. 2017; Feldmann et al. 2022). $\sigma ^2_{a^*}$ is simply a scaling factor that does not have a useful biological interpretation. It is important to note that the genomic best linear unbiased predictors (BLUPs) estimated by the model are unaffected by the scaling of the *G* matrix; they depend only on its structure (Feldmann et al. 2022). Therefore, we are not arguing against the use of the VanRaden relationship matrix, but rather showcasing that it is not suitable for the estimation of variance components through REML.

Other attempts at scaling the *G* matrix to unit variance include normalizing it to have average diagonal values equal to one (Forni et al. 2011; Vitezica et al. 2017), i.e., $\text {GN} = \frac{G}{tr(G)/n}$, where GN stands for normalized genomic relationship matrix, *tr*() refers to the trace of a matrix and *n* is the number of genotypes. However, as we exemplify in Supplementary File 1, it presents some bias. Similarly, Feldmann et al. (2022) described a relationship matrix based on average semivariance: $K_{\text {ASV}} = \frac{G}{\text {tr}(G)/(n-1)}$. This is conceptually different to *GN*, but numerically the differences are negligible.

Alternatively, Lehermeier et al. (2017) proposed the M2 method, which can estimate $\sigma ^2_a$ independently of the scaling of *G* matrix. This method allows to extract the actual additive variance from the marker effects and the covariance matrix between marker positions. However, they failed to account for the shrinkage of the estimated marker effects, causing a consistent underestimation of additive variance (proof available in Appendix 2). Thus, the first objective of this work is to propose a methodology able to extract additive variance from a given LMM without bias and compare it to existing methodologies.

Among the many applications of accurate variance components, we will further explore their use in simulations. Since genomics became commonplace in plant breeding, simulations have served as a test-bed for new methodologies. The seminal work for genomic selection (Meuwissen et al. 2001) relied heavily on simulations, and numerous other studies have also incorporated them (Jannink 2010; Wimmer et al. 2013; Endelman et al. 2014; Bernardo 2014; Hickey et al. 2014; Heslot et al. 2015; Neyhart et al. 2017; Technow et al. 2012; Faux et al. 2016; Jighly et al. 2019) due to their flexibility, speed and negligible cost compared to performing empirical experiments. However, simulations are only useful if they faithfully replicate the genetic control of the traits of interest; otherwise, any conclusion drawn from them will probably not hold in actual breeding programs. Therefore, it is important to first estimate variance components from empirical field trials and then use them as the target variances for the simulation (Ould Estaghvirou et al. 2013). Thus, minimizing bias in the estimation of $\sigma ^2_a$ and other variance components is critical to enable improved simulations.

Once the variance components are estimated, it is possible to start the simulation process that mimics the empirical trait (Daetwyler et al. 2013; Sun et al. 2011; Faux et al. 2016; Yuan et al. 2012). Several key criteria must be met. These include i) ensuring stable marker effects across generations, ii) adjusting marker effects to match an assumed trait architecture, iii) maintaining coherence among different genetic factors, iv) ensuring the variance of the simulated effects matches the target variance, and v) accounting for the standard error of the sample variance (SEV).

The SEV is often neglected but it is crucial for ensuring the accuracy and reliability of the simulation results. For instance, we can assume that the average variance of the location effect for the trait of interest $\sigma ^2_{L}$ is known. Nevertheless, in real field trials, the value of the location variance varies across different years. Therefore, forcing all years to have a location variance equal to $\sigma ^2_{L}$ would not be realistic. To address this issue, different years can have different values of the location variance, with the average across years being equal to $\sigma ^2_{L}$. This creates the problem of controlling how much the location variance should vary across years, which can be done through the SEV.

It is often of interest to assume correlated locations, making the effective sample size (ESS) different from the number of locations and altering the SEV value. A similar problem arises in most terms of the simulation, such as genetic effects in which the genotypes are correlated. Therefore, a generalized ESS is needed for controlling the SEV. To our knowledge, none of the available equations for calculating ESS or SEV applies to a generalized multivariate normal distribution, limiting their application in this context (Bayley and Hammersley 1946; Vallejos and Osorio 2014; Berger et al. 2014; Lin et al. 2007; Morita et al. 2008; Cogley 1999; Bretherton et al. 1999; Thiébaux and Zwiers 1984; Afyouni et al. 2019; Chelton 1983). Thus, the second objective of this work is to find a generalized ESS for SEV calculation.

In summary, the objectives of this work are to 1) find an improved method for estimating variance components from a LMM robust to the improper scaling of the *G* matrix, 2) develop a method to calculate the generalized ESS and SEV for any multivariate normal distribution and 3) build a simulation strategy that combines both advancements to meet all the quality criteria outlined previously and match reality as closely as possible.

## Materials and methods

**Fig. 1:**
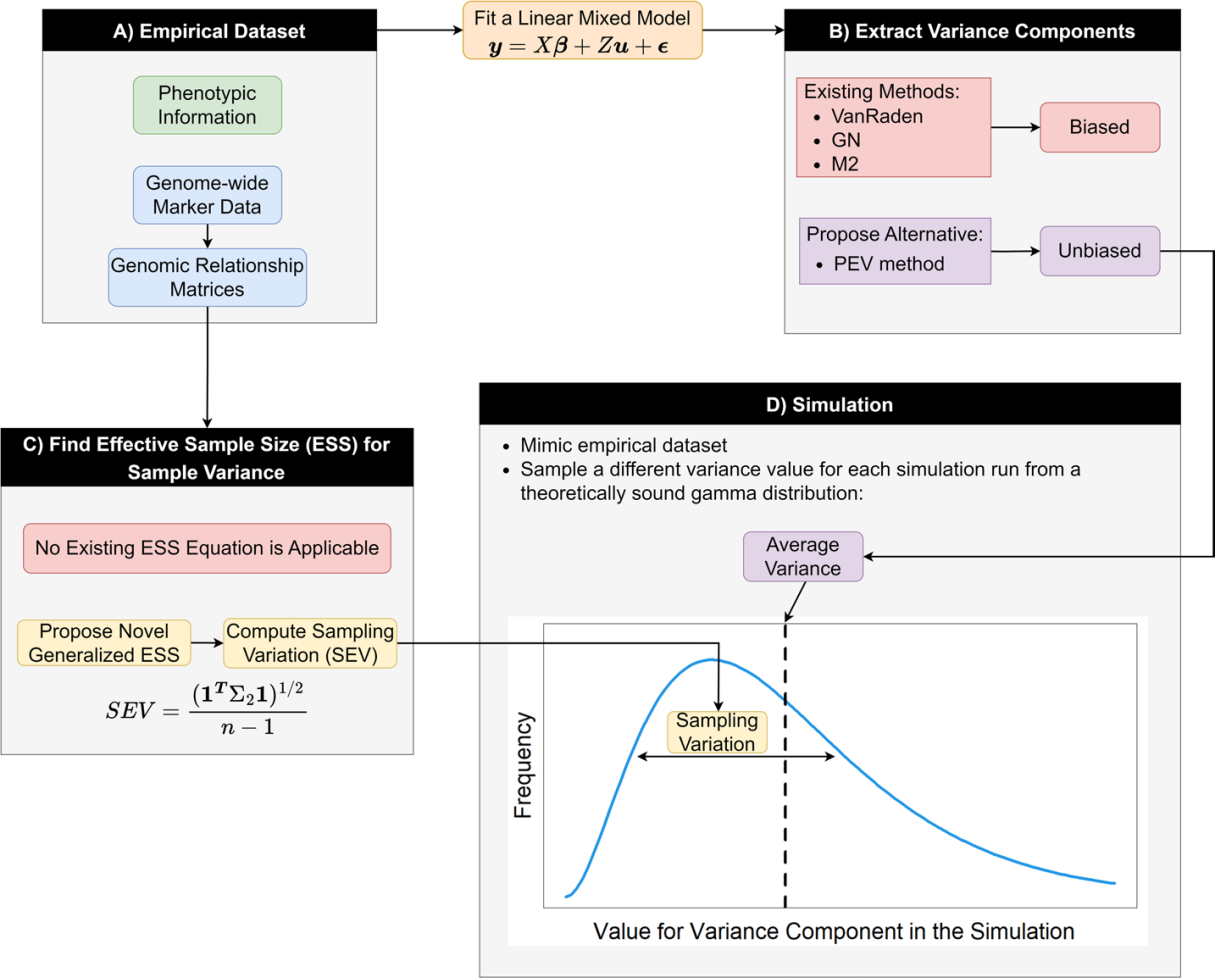
Overview of the study structure. Using an empirical dataset, we estimated genetic and non-genetic variance components, which were used as the baseline for simulation variance components. A novel equation for the standard error of the variance (SEV) was applied to control sampling variation across simulation runs. Detailed descriptions of all methods and equations are provided in the following sections

In Fig. 1 we have summarized the main modules of this paper, showcasing how they interact to enable more realistic simulations.

### Materials

In this section, we will explain the two empirical datasets we used in this work (Fig. 1A). We chose a highly homozygous oat dataset and a highly heterozygous spruce dataset. This allowed us to test the methodologies across varying degrees of heterozygosity, which significantly impacts variance estimation (Feldmann et al. 2022). Both datasets include several traits, ensuring consideration of varied trait architectures.

The oat dataset (Canales et al. 2021a) comprises 699 lines genotyped with 17,288 SNP markers after quality control and phenotyped in a randomized complete block design (RCBD) across three environments with three blocks each. All lines were almost completely homozygous and exhibited a strong population structure. The inbred nature of the lines ensures that they are not in Hardy–Weinberg equilibrium (HWE). Among the environments, two corresponded to different locations within the same year, and one to a different year. The latter was discarded to avoid the large variation across-year. Four traits were included in this study, yield, biomass, height, and heading days.

The spruce dataset (Beaulieu et al. 2014) contained 1722 lines with adjusted phenotypes summarizing the performance across two environments for three traits: diameter at breast height, density, and height. The lines were genotyped with 6930 SNP markers after quality control. In contrast to the oat dataset, population structure was weak, and 27.6% of the marker positions were heterozygous. We applied a corrected $\chi ^2$ test at each marker position to assess deviations from HWE (Graffelman 2010). Out of 6930 loci, 4480 were in HWE with a *p* value above 0.01, while 2450 showed significant deviations, exceeding the expected type I errors rate ($6930/100 = 69.3$). Thus, although the population is not fully in HWE, it remains close. It is important to note that this approach provides a broad assessment of whether a population is in Hardy–Weinberg equilibrium (HWE); however, it does not identify specific loci deviating from HWE due to the absence of a multiple-test correction. For example, applying a Bonferroni correction reveals that only 909 markers significantly diverge from HWE.

**Fig. 2:**
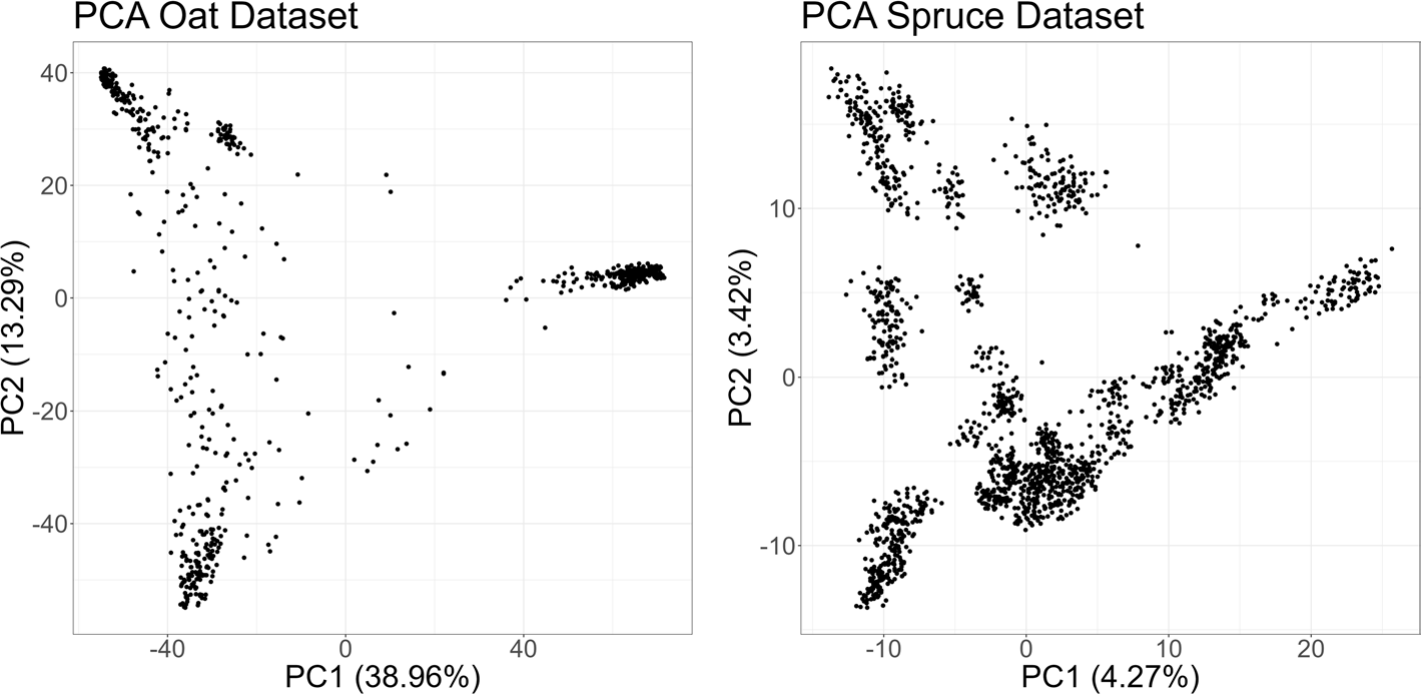
Genotypes are plotted based on their first two principal components (PC1, PC2) derived from the double-centered marker matrix for both datasets. The percentage of variance explained is shown in brackets next to each principal component

To further investigate the population structure, we conducted a principal component analysis on the double-centered (Gauch Jr et al. 2019) marker matrix of both datasets. Figure 2 displays individuals based on their first two principal components (PCs). In oat, these PCs account for over 50% of the variance, indicating strong population structure. In spruce, although some clusters are visually apparent, the first two principal components explain less than 10% of the variance, suggesting weak population structure and small genetic distances between clusters.

### Methods for variance component estimation

We used several methods to estimate additive variance (Fig. 1, B) and evaluated their accuracy. We tested variance estimation using the classical VanRaden (2008) relationship matrix, the GN matrix (the only unbiased estimator in Feldmann et al. (2022)), and the M2 method from Lehermeier et al. (2017), which was also shown to be unbiased in its original paper. However, as explained in the introduction, we believe that none of these methods reliably produce unbiased estimates. Therefore, we propose a new methodology based on prediction error variance (PEV) to address this issue.

#### VanRaden method

Let *M* be a matrix of marker scores, with *n* individuals in rows, *p* marker positions in columns and values within the matrix counting the number of copies of the minor allele in each position. Then, *Z* is the matrix obtained by column-centering *M*. This allows to compute the VanRaden (2008) matrix for diploid species as:

1 $$\begin{aligned} G = \frac{ZZ^T}{2 \sum _{j=1}^p p_j(1-p_j)} \end{aligned}$$

where $p_j$ is the minor allele frequency for marker *j*. The variance component estimation is done by fitting a mixed model to the data with a random effect for the additive values: ${\varvec{a}} \sim \text {MVN}({\varvec{0}}, G\sigma ^2_{a^*})$. $\sigma ^2_{a^*}$ will be used as the value for the variance component, i.e., it will be assumed that $\sigma ^2_{a^*} = \text {var}({\varvec{a}}) = \sigma ^2_a$.

The derivation of Eq. (1) and an explanation of why it relies on unrealistic assumptions are available in Appendix 1.

#### Normalized genomic relationship (GN) method

A normalized relationship matrix GN is obtained as in Forni et al. (2011):

2 $$\begin{aligned} \text {GN} = \frac{G}{\text {tr}(G)/n} \end{aligned}$$

The trace of a matrix is the sum of its eigenvalues (Jonsson 1982). Thus, $\text {tr}(G)/n$ is the average of the eigenvalues of the *G* covariance matrix and is a common way to extract the overall variance from a covariance matrix. However, as the off-diagonals are completely disregarded, this method is biased (see Supplementary File 1). An extreme example would involve all instances in the GN matrix having a variance of 1 and correlations of 1. This would cause all genotypes to have the same additive values and therefore $\sigma ^2_a = \text {var}({\varvec{a}})$ would be equal to zero even if $\text {tr}(\text {GN})/n = 1$.

The variance component estimation is done by fitting a mixed model to the data in which ${\varvec{a}} \sim \text {MVN}({\varvec{0}}, \text {GN}\sigma ^2_{a^*})$ and using $\sigma ^2_{a^*}$ as the estimate for the additive variance.

#### M2 method

Instead of directly relying on REML in a mixed model, additive variance can be alternatively estimated from the marker effects as described by Lehermeier et al. (2017):

3 $$\begin{aligned} \sigma ^2_a = \text {var}({\varvec{a}}) = \varvec{\beta }^T \Sigma _M \varvec{\beta } \end{aligned}$$

where $\beta$ is a vector of additive marker effects and $\Sigma _M$ is the covariance matrix between markers. This method directly accounts for linkage disequilibrium to address the limitations of VanRaden and GN methods. However, in Appendix 2 we argue that the M2 method is also unsuitable because it calculates the variance of the shrunk BLUPs rather than the actual population variance of the true breeding values. Furthermore, for large numbers of markers, computing $\Sigma _M$ can become unfeasible.

#### Prediction error variance (PEV) method

We propose this methodology to correct for the shrinkage in the M2 method, thereby eliminating the source of the bias. Furthermore, we will use an alternative implementation based on the genomic estimated breeding values (GEBVs) rather than on marker effects, massively reducing the computational burden. According to Henderson (1975), it is known that:

$$\begin{aligned} \text {cov}\begin{pmatrix} {\varvec{a}} \\ \varvec{{\hat{a}}} \end{pmatrix} = \begin{pmatrix} G\sigma ^2_{a^*} & G\sigma ^2_{a^*} - \text {PEV} \\ G\sigma ^2_{a^*} - \text {PEV} & G\sigma ^2_{a^*} - \text {PEV} \end{pmatrix} \end{aligned}$$

where $\varvec{{\hat{a}}}$ is a vector of estimated additive genotypic values (i.e., they are shrunk BLUPs) and PEV is the matrix of prediction error variance-covariance. $\text {cov}(\cdot )$ denotes the variance-covariance matrix of a random vector. Although $\text {var}(\cdot )$ is commonly used for this purpose, in our work, we reserve $\text {var}(\cdot )$ to indicate the sample variance of a vector, which is a scalar rather than a matrix. From this, we can express $\text {cov}({\varvec{a}})$ as a function of $\text {cov}(\varvec{{\hat{a}}})$ and PEV:

$$\begin{aligned} \text {cov}(\varvec{{\hat{a}}}) = G\sigma ^2_{a^*} - \text {PEV};\\ \text {cov}(\varvec{{\hat{a}}}) = \text {cov}({\varvec{a}}) - \text {PEV};\\ \text {cov}({\varvec{a}}) = \text {cov}(\varvec{{\hat{a}}}) + \text {PEV}; \end{aligned}$$

Therefore, the scalar $\sigma ^2_a = \text {var}({\varvec{a}})$ can be expressed as:

4 $$\begin{aligned} \small \sigma ^2_a = \text {var}({\varvec{a}}) = \text {var}(\varvec{{\hat{a}}}) + \frac{\text {tr}(\text {PEV})}{n} = \frac{\sum _{i=1}^n ({\hat{a}}_i - \bar{a})^2}{n} + \frac{\text {tr}(\text {PEV})}{n} = \frac{\sum _{i=1}^n {\hat{a}}_i^2}{n} + \frac{\text {tr}(\text {PEV})}{n} \end{aligned}$$

The value of $\sigma ^2_a$ is calculated from the variance of the shrunk BLUPs in $\varvec{{\hat{a}}}$ and the average PEV is added to compensate for the shrinkage. Please note that in the denominator of the sample variance of $\varvec{{\hat{a}}}$ we have used *n* instead of $n-1$ because the BLUPs are always mean-centered, which means that their population mean is known to be zero ($\bar{a} = 0$) and there is no need to expend a degree of freedom estimating it.

This method still requires extracting a scalar that summarizes the overall variance from the multivariate PEV matrix as the mean of its trace, which will incur in some bias as explained previously and exemplified in Supplementary file 1. However, we argue that the error incurred when calculating the average PEV is smaller than that caused by the scaling of GN. The reason is that the variances in PEV are always smaller or equal to those in $\text {GN}\sigma ^2_{a^*}$. As a result, if a similar relative error occurs when extracting the overall variance from PEV and $\text {GN}\sigma ^2_{a^*}$, a smaller absolute error will be incurred for the PEV matrix, minimizing bias. Furthermore, the PEV method accounts for linkage disequilibrium and marker effects, which are implicitly included in the vector of BLUPs, whereas the REML estimate of $\sigma ^2_{a^*}$ does not. It is also important to note that the PEV method relies on the REML estimate of $\sigma ^2_{a^*}$ and other variance components to construct the mixed model equations (Henderson 1975) necessary for computing the BLUPs and PEV matrix. Therefore, rather than an alternative to REML, the PEV method serves as a correction to REML estimates to incorporate linkage disequilibrium and the distribution of marker effects.

### Validation pipeline for variance estimation

To find which estimation method is better, we tested them in several models for both the oat and the spruce dataset. To that end, we applied a validation methodology similar to the one in Lehermeier et al. (2017). The following two-stage model was be used:

5 $$\begin{aligned} {\varvec{y}} = X_{L} {\varvec{L}} + X_{Block} \varvec{Block} + X_g{\varvec{g}} + \varvec{\epsilon } \end{aligned}$$

where ${\varvec{y}}$ is a vector of phenotypes, ${\varvec{L}}$ is a vector of fixed location effects, $\varvec{Block}$ is a vector of fixed block effects nested within location, ${\varvec{g}}$ is a vector fixed genotypic effects and $\varvec{\epsilon } \sim \text {MVN}({\varvec{0}}, R)$ is a vector of independent, identically distributed (i.i.d.) residuals within each location with heterogeneous variance across locations. $X_{L}$, $X_{Block}$ and $X_g$ are the design matrices of their corresponding effects. The ${\varvec{L}}$ and $\varvec{Block}$ effects were set as fixed because they did not present enough levels for a good variance component estimation by REML. Next, the genotypic best linear unbiased estimators ($\varvec{{\hat{g}}}$) were used as the response variable for a stage 2 model that partitions them into additive effects and residuals:

$$\begin{aligned} \varvec{{\hat{g}}} = {\varvec{a}} + \varvec{\epsilon } \end{aligned}$$

where ${\varvec{a}} \sim \text {MVN}({\varvec{0}}, K\sigma ^2_{a^*})$ is a vector of additive genotypic values and $\varvec{\epsilon } \sim \text {MVN}({\varvec{0}}, I\sigma ^2_\epsilon )$ is a vector of residuals. *K* can be the *G* or GN matrix as needed for each variance estimation method.

One important note about the notation we will use going forward: asterisks will be included in the subindex of the variance components (e.g., $\sigma ^2_{a^*}$) when they are just a scaling factor for a covariance structure without unit variance (such as *G*). The asterisk is used to indicate that the REML estimate is a non-interpretable value different from the actual variance component. Please note that for i.i.d. terms (e.g., $\sigma ^2_\epsilon$), the covariance matrix is the identity (scaled to unit variance) and in these cases, the asterisk is not included as the REML estimates are the actual variance component.

From the model assumption that the residuals are independent from the other model effects, we can state that, within the stage 2 model:

$$\begin{aligned} & \text {var}(\varvec{{\hat{g}}}) = \text {var}({\varvec{a}} + \varvec{\epsilon }) = \text {var}({\varvec{a}}) + \text {var}(\varvec{\epsilon });\\ & \sigma ^2_a = \text {var}({\varvec{a}}) = \text {var}(\varvec{{\hat{g}}}) - \text {var}(\varvec{\epsilon }) = \frac{\sum _{i=1}^n ({\hat{g}}_i - \bar{{\hat{g}}})^2}{n-1} - \sigma ^2_\epsilon \end{aligned}$$

This method of calculating $\sigma ^2_a$ is unbiased, as it involves subtracting the sample variance (an unbiased estimator) of the response variable and the REML estimation (an unbiased estimator) of the residual variance. This is only possible because there is a single random effect in the model. Thus, this methodology is useful for validation purposes in this simple model, but its applicability is limited outside this context. We can compare the VanRaden, GN, M2, and PEV methods for estimating $\sigma ^2_a$ against the unbiased estimation of $\sigma ^2_a = \frac{\sum _{i=1}^n ({\hat{g}}_i - \bar{{\hat{g}}})^2}{n-1} - \sigma ^2_\epsilon$, allowing us to identify the estimator with the lowest bias. Furthermore, in Appendix 5, we have also evaluated the bias of $\sigma ^2_a$ estimated in the framework of the natural and orthogonal interactions (NOIA) model (Vitezica et al. 2017).

It is important to clarify that, in this context, bias strictly refers to the error incurred when extracting the additive variance component from a given model. Bias resulting from an unsuitable model, such as one based on unrealistic assumptions, is beyond the scope of this work.

To study the effect of the experimental design, we also performed this analysis in an unbalanced subset of the oat dataset. We randomly discarded 75% of the observations in each location, yielding a highly unbalanced dataset. Then, we estimated the variance components using this unbalanced dataset and performed 20 repetitions to account for the influence of the random selection of observations. We compared the average variance values across repetitions for the different estimation methodologies.

### Generalized effective sample size

Here, we will introduce the equations we propose for computing sampling variation for Fig. 1C. It is well known that the standard errors in the estimation of sample mean (SEM) and sample variance (SEV) are heavily influenced by sample size. When the available data are comprised of *n* i.i.d. observations sampled from a normal distribution of variance $\sigma ^2$, the standard errors can be calculated as follows:

6 $$\begin{array}{*{20}l} {{\text{SEM}} = \frac{\sigma }{{n^{{1/2}} }}} \hfill \\ {{\text{SEV}} = \frac{{2^{{1/2}} \sigma ^{2} }}{{(n - 1)^{{1/2}} }}} \hfill \\ \end{array} {\text{ }}$$

However, correlated data often carry less information than i.i.d. samples, i.e., their effective sample size (ESS) is usually lower than the census count. For instance, the information carried by *n* observations that are perfectly correlated is equal to an ESS of one, as all observations after the first one are redundant. A lot of research is available in the literature for the ESS, but it mostly focuses on the sample mean and in autoregressive processes, linear models, and Bayesian statistics (Vallejos and Osorio 2014; Berger et al. 2014; Lin et al. 2007; Morita et al. 2008; Cogley 1999; Bretherton et al. 1999; Thiébaux and Zwiers 1984; Afyouni et al. 2019; Chelton 1983). Thus, as far as we are concerned, a generalized way of calculating ESS for sample variance is not available in the literature. Bayley and Hammersley (1946) show how it can be calculated in an autoregressive process, and our work could be seen as a generalization of it for any multivariate normal distribution. We have described how SEM, SEV and their associated effective sample sizes ($\text {ESS}_\mu$ for the mean and $\text {ESS}_{\sigma ^2}$ for the variance) can be calculated even in the presence of heteroscedasticity, heterogeneous correlations, and nonuniform means. For the sake of brevity, here we will just show the final equations, but a detailed mathematical derivation of them is available in Appendix 3. For any ${\varvec{X}} = \{X_1, X_2,\ldots , X_n\} \sim \text {MVN}(\varvec{\mu }, \Sigma )$:

7 $$\begin{array}{*{20}l} {{\text{SEM}} = \left( {\frac{{\overline{{\sigma ^{2} }} }}{{{\text{ESS}}_{\mu } }}} \right)^{{1/2}} = \frac{{({\mathbf{1}}^{\user2{T}} \Sigma {\mathbf{1}})^{{1/2}} }}{n}} \hfill \\ {{\text{SEV}} = \frac{{2^{{1/2}} \overline{{\sigma ^{2} }} }}{{({\text{ESS}}_{{\sigma ^{2} }} - 1)^{{1/2}} }} = \frac{{({\mathbf{1}}^{\user2{T}} \Sigma _{2} {\mathbf{1}})^{{1/2}} }}{{n - 1}}} \hfill \\ {\overline{{\sigma ^{2} }} = \frac{{tr(\Sigma )}}{n}} \hfill \\ {\Sigma _{2} [i,j] = 2(\Sigma _{1} [i,j])^{2} + 4\Sigma _{1} [i,j](\mu _{i} - \bar{\mu })(\mu _{j} - \bar{\mu })} \hfill \\ {\Sigma _{1} [i,j] = \Sigma [i,j] + {\text{SEM}}^{2} - \frac{{\Sigma [i,1:n]{\mathbf{1}}}}{n} - \frac{{\Sigma [j,1:n]{\mathbf{1}}}}{n}} \hfill \\ {{\text{ESS}}_{\mu } = \frac{{tr(\Sigma )}}{{{\mathbf{1}}^{\user2{T}} \Sigma {\mathbf{1}}}}n} \hfill \\ {{\text{ESS}}_{{\sigma ^{2} }} = \frac{{2(\overline{{\sigma ^{2} }} )^{2} (n - 1)^{2} }}{{{\mathbf{1}}^{\user2{T}} \Sigma _{2} {\mathbf{1}}}} + 1} \hfill \\ \end{array}$$

It is important to clarify that, in Eq. (7), $\Sigma$, $\Sigma _1$ and $\Sigma _2$ refer to different matrices. $\Sigma _1$ is derived from $\Sigma$ and $\Sigma _2$ is derived from $\Sigma _1$ and $\varvec{\mu }$. To validate the proposed methods, we tested them across various scenarios, incorporating autoregressive covariance structures and additive and dominance relationship matrices with different means. We compared the SEM and SEV calculated as proposed in Eq. (7) with: i) the standard error of the mean and variance estimated under the assumption of i.i.d. observations with homogeneous means (Eq. 6) and ii) empirical standard errors, calculated as the standard deviation of the sample mean and sample variance estimated from $10^5$ samples drawn from the corresponding multivariate normal distributions.

### Simulation strategies

We compared the two most common simulation strategies to our proposed methodology, which is based on the PEV variance component estimation and the usage of $\text {ESS}_{\sigma ^2}$ to introduce sampling variance as outlined in Fig. 1D. First, we fitted a model to the oat dataset for yield to extract the variance components used as targets for the simulation:

8 $$\begin{aligned} {\varvec{y}} = X_{L} {\varvec{L}} + X_{Block} \varvec{Block} + Z_a{\varvec{a}} + Z_{aL}{{\varvec{a}}}{{\varvec{L}}} + \varvec{\epsilon } \end{aligned}$$

Where all effects are the same as in Eq. (5) where applicable, ${\varvec{a}} \sim \text {MVN}({\varvec{0}}, G\sigma ^2_{a^*})$ is a vector of additive genotypic values and ${{\varvec{a}}}{{\varvec{L}}}$ is a vector of additive by location interaction with the following covariance structure:

$$\begin{aligned} {{\varvec{a}}}{{\varvec{L}}} \sim \text {MVN}({\varvec{0}}, \begin{pmatrix} \sigma ^2_{aL1^*} & 0 \\ 0 & \sigma ^2_{aL2^*} \end{pmatrix}\otimes G) \end{aligned}$$

where $\otimes$ refers to the Kronecker product. We assumed the different locations were independent to reduce the number of parameters the model needed to estimate and to facilitate the estimation of the overall variance component from the interaction terms. $\varvec{\epsilon }$ represents the vector of residuals with heterogeneous variance across locations:

$$\begin{aligned} \varvec{\epsilon } \sim \text {MVN}\left({\varvec{0}}, \begin{pmatrix} \sigma ^2_{\epsilon L1} & 0 \\ 0 & \sigma ^2_{\epsilon L2} \end{pmatrix}\otimes I\right) \end{aligned}$$

The design matrices for their corresponding effects are $X_L$, $X_{Block}$, $Z_a$, and $Z_{aL}$. Non-additive effects such as dominance and epistasis were excluded for the sake of simplicity. We estimated all variance components of random effects using REML and by the PEV method. For interaction effects and residuals with heterogeneous variances across locations, the overall variance was calculated as the average of the variance of the two locations (a weighted mean was unnecessary due to the balanced experimental design). The variance of the fixed location effect was calculated as $\text {var}({\varvec{L}}) = \text {var}(X_{L} \hat{{\varvec{L}}})$, where $\hat{{\varvec{L}}}$ is the vector of best linear unbiased estimators. The variance of the block effect was calculated similarly within each location and the average across locations was taken as the overall variance.

For all simulations, we randomly selected a subset of 50 genotypes and used their empirical genotypic information. We simulated field trials consisting of 5 locations with 3 blocks each, arranged in RCBD. We assumed that the locations had a compound symmetry covariance structure, with homogeneous variance and a common correlation of 0.3. These positive correlations reflect a similarity between locations, which is typical in breeding programs targeting specific environmental profiles. All effects in Eq. (8) were simulated, and the phenotype was calculated as the sum of all of them. We performed 500 repetitions for each simulation type.

#### MVN simulation: not marker-based

All effects were sampled from their corresponding normal distributions, which is the reason why we will refer to this approach as the multivariate normal (MVN) one:

9 $$\begin{array}{*{20}l} {\user2{L}\sim {\text{MVN}}\left( {{\mathbf{0}},K_{e} \sigma _{{L^{*} }}^{2} } \right)} \hfill \\ {\user2{Block}\sim N\left( {0,\sigma _{{Block}}^{2} } \right)} \hfill \\ {\user2{a}\sim {\text{MVN}}\left( {{\mathbf{0}},G\sigma _{{a^{*} }}^{2} } \right)} \hfill \\ {\user2{aL}\sim {\text{MVN}}\left( {{\mathbf{0}},K_{e} \otimes G\sigma _{{aL^{*} }}^{2} } \right)} \hfill \\ {\varepsilon \sim N\left( {0,\sigma _{\varepsilon }^{2} } \right)} \hfill \\ \end{array}$$

where $K_e$ is the covariance structure assumed for the locations (common variance with unit value and correlations of 0.3). The values for all variance components were the REML estimates from the model in Eq. (8). The PEV estimate of the variances was not used because it does not compensate for the inadequate scaling of the *G* matrix, while the REML estimate does.

#### Fixed simulation: marker-based, fixed variance components

In this methodology, all effects were initially simulated at the marker level with arbitrary variance. In the second step, the effects were scaled to match the target variance exactly. For this reason, we will refer to this simulation as the ‘Fixed’ approach going forward. The target variance values were obtained using the PEV method of variance estimation as explained in Sect. 2.4.

Additive effects were based on 2500 randomly selected markers that acted as quantitative trait loci (QTL). It is important to note that, since we were simulating a single field trial, there is no drawback in setting the markers to be the actual QTL. However, for simulations spanning several generations, it would be important to instead treat the QTL as positions in linkage disequilibrium with the markers, which would allow to consider the variation in linkage disequilibrium patterns across generations due to recombination during meiosis (Daetwyler et al. 2013; Sun et al. 2011; Faux et al. 2016; Yuan et al. 2012).

Non-scaled additive marker effects ($\varvec{\beta ^*}$) for the 2500 QTL were sampled from a gamma distribution with shape = 0.4, scale = 1.66. These parameters are commonly used in the literature, such as in Meuwissen et al. (2001) and Technow et al. (2012). To avoid the minor allele always having a positive effect, we randomly multiplied the additive marker effects by 1 or $-1$ with equal probability. Non-scaled additive values ($\varvec{a^*}$) were obtained as $\varvec{a^*} = M\varvec{\beta ^*}$, where *M* refers to the matrix of numeric marker scores. Next, we scaled $\varvec{a^*}$ and $\varvec{\beta ^*}$ to the desired target variance, which can easily be done by dividing them by their standard deviation and multiplying by the target standard deviation:

$$\begin{aligned} & \varvec{\beta } = \varvec{\beta ^*}\frac{\sigma _a}{sd(\varvec{a^*})}\\ & {\varvec{a}} = M\varvec{\beta } \end{aligned}$$

where $\varvec{\beta }$ are the scaled marker effects and $\sigma _a$ is the PEV estimate for the standard deviation of the additive effect. ${\varvec{a}}$ contains the scaled additive values, i.e., $sd({\varvec{a}}) = \sigma _a$.

For the simulation of the additive by location interaction, the aim was to obtain a matrix *AxL* with genotypes in the rows and locations in the columns, where the elements correspond to the additive by location interaction values. The first step was defining a matrix $M_a$ with dimensions corresponding to *n* individuals in the rows and *p* QTL in the columns. This matrix contains the marker scores in *M* multiplied by their corresponding marker effects in $\varvec{\beta }$, i.e.,:

$$\begin{aligned} M_a = M \begin{pmatrix} \beta _1 & 0 & \dots & 0 \\ 0 & \beta _2 & & 0 \\ \vdots & & \ddots & \vdots \\ 0 & 0 & \dots & \beta _p \end{pmatrix} \end{aligned}$$

Next, 100 environmental covariates for each location were sampled from $\text {MVN}({\varvec{0}}, K_e)$ and the results were stored in a matrix ($L_{\text {cov}}$) with the environmental covariates in the rows and the locations in the columns. A matrix $B_{aL}$ with dimensions $p \times t$ (number of QTL times number of environmental covariates) was created and its elements were sampled from a standard normal distribution. $B_{aL}$ contains the intensity of interaction between additive effects and environmental covariates. The non-scaled additive by location interaction matrix $AxL^*$ was obtained by multiplying each marker effect in $M_a$ by each environmental covariate in $L_{\text {cov}}$ and their interaction intensity in $B_{aL}$ and adding up the corresponding values for each genotype-location combination. In matrix notation:

$$\begin{aligned} AxL^* = M_a B_{aL} L_{\text {cov}} \end{aligned}$$

Subsequently, the $AxL^*$ was double-centered to remove any main effects and leave only the interaction terms. Finally, the double-centered matrix ($AxL^*_c$) was scaled to the target variance as:

$$\begin{aligned} AxL = AxL_c^* \frac{\sigma _{aL}}{sd(AxL_c^*)} \end{aligned}$$

where $sd(AxL_c^*)$ refers to computing the standard deviation of all elements in the matrix and $sd(AxL) = \sigma _{aL}$.

The non-scaled location main effects $\varvec{L^*}$ were calculated as the sum of the column means of $AxL^*$. Then, the scaled effects ${\varvec{L}}$ were calculated as:

$$\begin{aligned} {\varvec{L}} = \varvec{L^*} \frac{\sigma _{L}}{sd(\varvec{L^*})} \end{aligned}$$

Finally, within each location, the non-scaled block effects $\varvec{Block^*}$ and residuals $\varvec{\epsilon ^*}$ were sampled from a standard normal distribution and scaled to the target variance to obtain:

$$\begin{aligned} & \varvec{Block} = \varvec{Block^*}\frac{\sigma _{Block}}{sd(\varvec{Block^*})}\\ & \varvec{\epsilon } = \varvec{\epsilon ^*}\frac{\sigma _{\epsilon }}{sd(\varvec{\epsilon ^*})} \end{aligned}$$

where $\varvec{Block}$ and $\varvec{\epsilon }$ are the scaled block and residual values.

#### ESS simulation: marker-based, variable variance components

This simulation was performed similarly to the previous one, except for the step for scaling the effects to the target variance. In the previous case, scaling was done by dividing all values by their standard deviation and multiplying by the target standard deviation estimated from the empirical data. Conversely, in this scenario, a variable scaling factor was used to account for sampling variance. This approach uses the fact that sample variance follows a gamma distribution with a mean equal to the population variance and a standard deviation equal to SEV. As many effects are not i.i.d., calculating SEV requires considering its $\text {ESS}_{\sigma ^2}$. For this reason, we will refer to this simulation as the ‘ESS’ approach going forward.

For the additive effects, the population variance is the desired target variance $\sigma ^2_a$ (estimated through the PEV method) and, as shown in Eq. (7), the standard deviation SEV can be calculated from $\Sigma = \text {GN}\sigma ^2_a$, $\varvec{\mu } = {\varvec{0}}$ and *n* equal to the number of genotypes. Please note that the *GN* matrix is used in the calculation of $\Sigma$ because having a diagonal with unit average ensures that, in Eq. (7), $\bar{\sigma ^2} = \frac{\text {tr}(\Sigma )}{n} = \frac{\text {tr}(\text {GN})}{n}\sigma ^2_a = \sigma ^2_a$. After computing SEV, we can sample a variance value $s^2_a$ from a gamma distribution of mean $\sigma ^2_a$ and variance $\text {SEV}^2$, i.e., $s^2_a \sim \Gamma (\mu = \sigma ^2_a, \sigma ^2 = \text {SEV}^2)$. For ease of use, it is possible to find the scale ($\theta$) and shape (*k*) parameters of the gamma distribution from its mean and variance as follows: $\theta = \frac{\sigma ^2}{\mu } = \frac{\text {SEV}^2}{\sigma ^2_a}$ and $k = \frac{\mu ^2}{\sigma ^2} = \frac{\sigma ^4_a}{\text {SEV}^2}$. Finally, the square root of the sampled $s^2_a$ can be used as a variable scaling factor when scaling the marker effects:

$$\begin{aligned} \varvec{\beta } = \varvec{\beta ^*} \frac{s_a}{sd(\varvec{a^*})}; s^2_a \sim \Gamma \left( \theta = \frac{\text {SEV}^2}{\sigma ^2_a}, k = \frac{\sigma ^4_a}{\text {SEV}^2}\right) \end{aligned}$$

In summary, as $s^2_a$ is a random sample, $\text {var}({\varvec{a}}) = \text {var}(M\varvec{\beta }) = s^2_a$ will have a different value in each simulation run, but they will be centered around the target $\sigma ^2_a$, following a theoretically sound distribution with standard deviation equal to SEV.

The same can be done for all the other effects, with $\Sigma$ for the calculation of SEV being the assumed covariance matrices for each effect (see Eq. 9) and *n* is the number of rows or columns in $\Sigma$. For the i.i.d. block and residual effects, *SEV* can be calculated as in Eq. (6). For a more detailed overview, we provide the code to perform this simulation in Supplementary File 2.

#### Validation pipeline for the simulation strategies

The three simulation strategies (MVN, Fixed, and ESS) were repeated 500 times using the variance components extracted from the oat dataset in Eq. (8) as the target. The average variance of each effect across iterations for each simulation type was computed. The theoretical distribution of this average is known (see Appendix 4), allowing us to check if the average variance across iterations falls within a 95% confidence interval of the expected value. This helps to determine if the variances obtained by the simulations significantly differ from the desired target value, indicating potential bias in the simulation. Additionally, we checked if the dispersion of the variance values across iterations matches the expected distribution to validate the inclusion of sampling variance in the simulations.

In summary, we evaluated the simulation strategies based on their ability to be unbiased and accurately include sampling variation.

## Results

### Comparison of methods for variance component estimation

The results in Table 1 confirm that the VanRaden, GN and M2 methods are biased, while the PEV methodology was mostly unbiased, exhibiting the lowest error by far. In the oat dataset with a balanced experimental design, the root mean square error (RMSE) for variance values with respect to the unbiased estimation was 0.00316, 0.340, 0.473, and 0.132 for the PEV, VanRaden, GN, and M2 methods, respectively. A similar but less extreme pattern was present in the spruce dataset, with RMSE values of 0.00254, 0.0511, 0.0483, and 0.460 for PEV, VanRaden, GN, and M2. When unbalance was introduced in the oat dataset, the error for VanRaden, GN, and M2 methods increased substantially, but it remained extremely low for PEV. The RMSE values were 0.00441, 0.581, 1.80, and 0.333 for PEV, VanRaden, GN, and M2, respectively. In summary, the largest relative error for the PEV methodology across all traits and scenarios is 0.5%. Conversely, other methods presented much larger errors, with the largest one being a 200% error for the *GN* methodology in the unbalanced dataset for the biomass trait.

The genomic relationship matrices used previously assumed HWE, which was not realistic, particularly for the oat dataset. To assess the impact of this, we repeated the analyses using the genomic relationship matrix from the NOIA model (Vitezica et al. 2017), which does not assume HWE (See Appendix 5, Table 4). This resulted in a RMSE of 0.404, 1.63 and 0.0476 for the balanced oat, unbalanced oat, and spruce datasets, respectively, when using REML estimates of additive variance. With PEV estimates, RMSE was significantly lower, ranging from 0.0113 to 0.00280.

All values above apply for the estimation of the additive variance, but we also tested the PEV method for the estimation of genotypic variance when the model had an i.i.d. genotypic random effect, i.e., ${\varvec{g}} \sim \text {MVN}({\varvec{0}}, I\sigma ^2_g)$. The results for this analysis are available in Appendix 6, Table 5, and they show that the PEV method had negligible differences with the unbiased estimate for balanced designs (RMSE $\approx 10^{-4}$), but it presented a small error for the unbalanced dataset, with relative errors ranging between 0.1% and 7% depending on the trait (RMSE $= 0.00458$).

**Table 1:** Comparison of the variance component estimation between the different methods in the oat and spruce datasets

Dataset	Experimental design	Trait	$\sigma ^2_{\text {Unbiased}}$	$\sigma ^2_{\text {PEV}}$	$\sigma ^2_{\text {VanRaden}}$	$\sigma ^2_{\text {GN}}$	$\sigma ^2_{M2}$
Oat	Balanced	Yield	1.000	1.004	0.554	0.983	0.897
Oat	Balanced	Biomass	1.000	0.998	0.752	1.333	0.781
Oat	Balanced	Height	1.000	1.004	1.064	1.885	0.901
Oat	Balanced	Heading days	1.000	1.000	0.557	0.987	0.964
Oat	Unbalanced	Yield	1.000	0.997	1.542	2.729	0.692
Oat	Unbalanced	Biomass	1.000	0.995	1.696	3.005	0.498
Oat	Unbalanced	Height	1.000	0.995	1.599	2.833	0.715
Oat	Unbalanced	Heading days	1.000	0.997	1.463	2.590	0.874
Spruce	Balanced	Diameter	1.000	1.000	1.000	1.006	0.467
Spruce	Balanced	Height	1.000	0.996	0.943	0.947	0.542
Spruce	Balanced	Density	1.000	1.000	0.933	0.935	0.623

For the unbalanced datasets, the average variance across 20 repetitions of randomly dropping observations from the full dataset are presented. The column for $\sigma ^2_{\text {Unbiased}}$ displays the difference between the variance of the response variable and residual variance. $\sigma ^2_{\text {VanRaden}}$ contains the REML estimation of the variances when using *G* as covariance structure of the additive effects, $\sigma ^2_{\text {GN}}$ estimates variance using GN as covariance structure of the additive effects, $\sigma ^2_{M2}$ estimates variance using M2 method and $\sigma ^2_{\text {PEV}}$ estimates the variance component with the PEV method. For ease of interpretation, the variance estimates in each row have been standardized in such a way that the value of $\sigma ^2_{\text {Unbiased}}$ is equal to one

### Validation of the proposed effective sample size for mean and variance

The results in Table 2 show the results of the SEM and SEV estimates for several normally distributed random variables. We compared the SEM and SEV wrongly assuming i.i.d. random variables ($\text {SEM}_{iid}$ and $\text {SEV}_{iid}$, computed as in Eq. 6), using the generalized SEM and SEV described in Eq. (7) ($\text {SEM}_{g}$ and $\text {SEV}_{g}$) and empirically ($\text {SEM}_{e}$ and $\text {SEV}_{e}$). The latter were calculated as the standard deviation of the sample mean and sample variance estimated across $10^5$ samples from the corresponding multivariate normal distributions. The data and code that generate the results in Table 2 are available in Supplementary Files 3 and 4.

Most of the scenarios tested (first seven rows in Table 2) are based on an autoregressive process of order one with different values of autocorrelation ($\rho$). The vector of means was sampled from a standard normal distribution to ensure that our methods are effective for heterogeneous means, i.e., ${\varvec{X}} \sim \text {MVN}(\varvec{\mu _{N(0,1)}}, \Sigma _{\text {AR}1(\rho )})$, where $\varvec{\mu _{N(0,1)}} \sim N(0,1)$ and $\Sigma _{\text {AR}1(\rho )}$ is the correlation matrix of the autoregressive process. It is important to note that using the correlation matrix as a variance-covariance matrix is not problematic because correlation and covariance are equivalent when all variables have a variance equal to one.

Two non-autoregressive scenarios were tested to ensure the robustness of our equations for ESS under non-autoregressive covariance structures: ${\varvec{X}} \sim \text {MVN}({\varvec{0}}, G)$ and ${\varvec{X}} \sim \text {MVN}({\varvec{0}}, Gd)$, where ${\varvec{0}}$ represents a vector of zero means, *G* is the additive genomic relationship matrix (VanRaden 2008) and *Gd* is the dominance genomic relationship matrix (Vitezica et al. 2013). Both matrices exhibit heteroscedasticity with heterogeneous correlation and were derived from genome-wide molecular markers in the oat dataset.

The results in Table 2 confirm that the values of $\text {SEM}_{iid}$ and $\text {SEV}_{iid}$ do not match reality for correlated observations. In contrast, the theoretical values for $\text {SEM}_{g}$ and $\text {SEV}_{g}$ match the empirical ones ($\text {SEM}_{e}$ and $\text {SEV}_{e}$) in all scenarios. The minor discrepancies observed are caused by the empirical values being estimated with a large, yet finite, sample size, i.e., the true SEM and SEV values are $\text {SEM}_{g}$ and $\text {SEV}_{g}$; while $\text {SEM}_{e}$ and $\text {SEV}_{e}$ are only empirical estimates with very low error.

**Table 2:** Theoretical and empirical values of the standard error of the mean (SEM) and the sample variance (SEV) are presented from correlated samples

${\mu }$	$\Sigma$	$\text {SEM}_{iid}$	$\text {SEM}_{g}$	$\text {SEM}_{e}$	$\text {SEV}_{iid}$	$\text {SEV}_{g}$	$\text {SEV}_{e}$
$\varvec{\mu } \sim N(0,1)$	$\text {AR}1(\rho = -1)$	0.100	0.000	0.000	0.142	1.434	1.432
$\varvec{\mu } \sim N(0,1)$	$\text {AR}1(\rho = -0.9)$	0.100	0.024	0.024	0.142	0.485	0.487
$\varvec{\mu } \sim N(0,1)$	$\text {AR}1(\rho = -0.1)$	0.100	0.091	0.091	0.142	0.265	0.266
$\varvec{\mu } \sim N(0,1)$	$\text {AR}1(\rho = 0)$	0.100	0.100	0.100	0.142	0.249	0.249
$\varvec{\mu } \sim N(0,1)$	$\text {AR}1(\rho = 0.1)$	0.100	0.110	0.110	0.142	0.244	0.245
$\varvec{\mu } \sim N(0,1)$	$\text {AR}1(\rho = 0.9)$	0.100	0.415	0.415	0.142	0.391	0.391
$\varvec{\mu } \sim N(0,1)$	$\text {AR}1(\rho = 1)$	0.100	1.000	0.997	0.142	0.000	0.000
$\varvec{\mu } = {\varvec{0}}$	*G*	0.038	0	0	0.054	0.633	0.634
$\varvec{\mu } = {\varvec{0}}$	$G_d$	0.038	0.710	0.709	0.054	0.177	0.176

The initial seven rows detail autoregressive processes of order one with varying autocorrelation ($\rho$) levels and nonzero means derived from a standard normal distribution. The last two rows correspond to two genomic relationship matrices, an additive matrix (*G*) and a dominance matrix ($G_d$), both characterized by zero means. $\text {SEM}_{iid}$ denotes the SEM assuming i.i.d. observations; $\text {SEM}_{g}$ represents the generalized SEM outlined in this work; $\text {SEM}_{e}$ is the empirical SEM; $\text {SEV}_{iid}$ indicates the SEV assuming i.i.d. and observations with homogeneous means; $\text {SEV}_{g}$ describes the generalized SEV introduced in this work; $\text {SEV}_{e}$ is the empirical SEV

### Ability of the different simulation approaches to control variance components

**Fig. 3:**
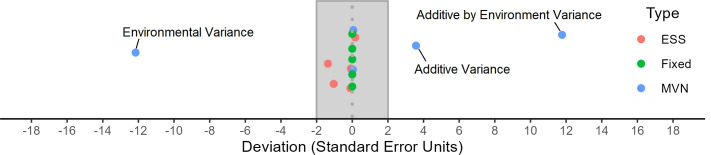
Deviation of the average variance of the simulated effects from the assumed population variance across the 500 simulation repetitions expressed in units of standard error of the mean. The points correspond to the different variance components for the three simulation strategies (in different colors). If the simulation variances are unbiased and distributed as expected in theory, they would be centered around zero and with deviations between – 2 and 2 (highlighted with a gray rectangle) with a probability of approximately 0.95. All outliers outside this range are labeled and they belong to the simulation strategy of sampling from multivariate normal distributions. More details about how we calculated the standard error and built the confidence interval are available in Appendix 4

In a simulation, the actual variance of simulated terms should not significantly differ from the assumed population variance. To assess this, Fig. 3 shows the average deviation of simulated variances from the assumed population variance across 500 repetitions. A deviation of zero indicates that the simulated variance matches the target population variance exactly. Deviations within the gray rectangle in Fig. 3 suggest that, while the simulated variances differ slightly from the assumed population variance, they are not significantly different, which is expected for a random sample. Deviations outside this range indicate that the simulated variances are significantly different from the population variance, leading to biased simulations. More details on the construction of the confidence interval are provided in Appendix 4.

The MVN simulation presented a strong dispersion, with the environmental, additive and additive by environment variances being significantly different from the desired target variance. Conversely, the fixed simulation always had exactly the desired value for the variance, i.e., it was unbiased but neglected sampling variance. Finally, the ESS simulation presented some dispersion (it accounted for sampling variance), but all values were within the 95% confidence interval, indicating that the simulated values were not significantly different from the desired target (i.e., the simulation was unbiased).

## Discussion

### Comparison of methods for variance component estimation

To estimate $\sigma ^2_{a}$ from field trials, the first step is fitting a model, commonly a LMM. This allows to partition phenotypic variance into its underlying components, such as $\sigma ^2_{a}$. However, if the model is based on unrealistic assumptions, $\sigma ^2_{a}$ may not accurately reflect the additive variance in the population, resulting in bias. This issue is discussed extensively by Chen (2014), Chen (2016), de Los Campos et al. (2015), but falls outside the scope of this work. Moreover, extracting $\sigma ^2_a$ from a given LMM is complex and can introduce further bias. In this work, we demonstrate that existing methods show substantial bias for complex traits and propose a PEV-based approach that minimizes it.

The REML estimation of $\sigma ^2_{a^*}$ when the *G* or GN matrices are assumed as covariance structures is unbiased in the sense that it scales the *G* or GN matrices to the most likely covariance matrix for the random effect. However, as shown in Table 1, interpreting $\sigma ^2_{a^*}$ as the variance component of the additive effect would be erroneous because neither *G* nor GN have unit variance. This contrasts with the results of Feldmann et al. (2022), in which GN performed very well. In that study, GN was tested in both simulations and empirical datasets. In the latter, they simply tested several methods for variance estimation, but they did not provide an unbiased estimate of the additive variance as a baseline for comparison. Therefore, their analyses in empirical datasets did not determine how accurate the GN method is.

Conversely, in the simulated data, they compared the $\sigma ^2_{a^*}$ variance estimated with GN to the true additive variance ($\sigma ^2_{a}$), and GN was unbiased. However, not many details about the simulation were provided in Feldmann et al. (2022). For instance, we can speculate that, when they simulated the marker scores, they may have not explicitly included correlations between them (i.e., linkage disequilibrium), which, as shown by Lehermeier et al. (2017), have a massive impact on the ability of the relationship matrix to be correctly scaled.

In contrast, in our work, we prove that the GN method is biased in two ways. Firstly, in Supplementary File 1, we demonstrate that the scaling factor used to generate GN (the average of the diagonal values in *G*) does not reliably capture the overall variation in *G* and therefore is unable to scale GN to unit variance. As a result, the GN method of variance estimation cannot be unbiased. Secondly, we tested the GN method in empirical datasets with varying levels of heterozygosity, population structure, balance in the experimental design, and trait architectures. In all circumstances, GN was biased, especially under low heterozygosity, unbalanced experimental designs and absence of HWE.

To further support this claim, we repeated the analysis with the additive genomic relationship matrix from the NOIA model (Vitezica et al. 2017). This matrix is similar to GN (it is scaled to have an average value for its main diagonal equal to one) but has the advantage of not requiring the assumption of HWE for the calculation of its covariance structure. The results from Table 4 in Appendix 5 show that the REML estimates when using this matrix were very similar to the error-prone GN.

The M2 method (Lehermeier et al. 2017) is extremely interesting because it bypasses the problem of scaling the *G* matrix. Equation (3) shows that additive variance is the sum of the variances of the marker scores plus twice their covariances, scaled by their corresponding marker effects. That way, strong linkage disequilibrium between two markers (positive covariance) increases the additive variance only if these two markers have substantial marker effects. This reflects that the distribution of marker effects along the genome conditions how much impact the linkage disequilibrium has on the additive variance. Therefore, as seen in Lehermeier et al. (2017), a good *G* matrix scaling needs to consider the linkage disequilibrium, which appears to be possible only if the distribution of marker effects along the genome is known. As a result, it seems to us that finding an unbiased scaling for the *G* matrix *a priori* is impossible, i.e., we cannot properly scale *G* before fitting the model and finding the marker effects. This is further supported by the fact that, in Table 1, the variance estimates for a given relationship matrix presented different errors for different traits, which means that a good scaling of the *G* matrix would need to be trait-specific. As a result, the REML estimates of the additive variance component cannot be unbiased regardless of which genomic relationship matrix is used as a covariance structure, explaining the poor performance of the VanRaden and GN methods.

While the M2 method accounts for linkage disequilibrium, it simply represents the variance of the additive BLUPs, as proven in Appendix 2. Due to the shrinkage of the BLUPs, their variance is smaller than the true population variance that we aim to estimate, resulting in a biased estimation. In Lehermeier et al. (2017), a dataset with high replication (4 replicates) and very high heritability (around 0.9), minimized the effect of shrinkage. This explains why the M2 method performed well for them but consistently underestimated additive variance in this work.

To solve this problem, we described the PEV method, which can be viewed as the M2 method with an added correction factor to compensate for shrinkage. Furthermore, the M2 method requires the computationally intensive covariance matrix between markers. Conversely, the PEV method is instead based on the genotypic additive values, which are much more computationally efficient, and carry equivalent information to the marker effects as shown in Appendix 7. Thus, the PEV method can still implicitly capture the effect of the linkage disequilibrium. As shown in Table 1, this allows the PEV method to present negligible bias when estimating the additive variance, making it by far the most accurate method we have encountered. This can significantly improve the accuracy of the study of the genetic control of traits, calculation of heritability, retrieval of target variances for simulations, and more.

### Implications of the proposed generalized effective sample size

As highlighted in Table 2, the generalized SEM and SEV described in this work perfectly match empirical values and are robust to different covariance structures and vectors of means. The equation for SEM is equivalent to the one described in Griffith (2008), but here we show that it holds for any multivariate normal distribution. Conversely, the equation for SEV was not previously described and it supposes a generalization of the equation in Bayley and Hammersley (1946) from an autoregressive process to any multivariate normal distribution, which is essential for its application in quantitative genetics.

Furthermore, SEM, SEV, and their associated ESS have some interesting properties that broaden their potential applications. $\text {ESS}_\mu$ and $\text {ESS}_{\sigma ^2}$ are insensitive to the multiplication of $\Sigma$ by a scalar if the vector of means is zero (which is always true for random effects). For instance, this results in the value of ESS being the same for $\Sigma = G$ or $\Sigma = G\sigma ^2_{a^*}$, making ESS an interesting tool for characterizing a population even if the value of their variance component is unknown.

Furthermore, both SEM and SEV (and their associated ESS) are sensitive to the degree of replication of the experiment, i.e., the SEM or SEV calculated with $\Sigma$ can differ from that computed from $Z\Sigma Z^T$, where *Z* is the design matrix of the experiment.

This sensitivity suggests potential applications for optimizing the training set in genomic selection. Coincidentally, as seen in Eq. (7), $\text {SEM}^2 = \frac{{\varvec{1}}^T \Sigma {\varvec{1}}}{n^2}$, which is the average of all elements in $\Sigma$ and is exactly equivalent to the Avg_GRM_self optimization criterion described in Fernández-González et al. (2023), aimed at maximizing training set diversity.

This makes sense, as minimizing SEM ensures that the average of all BLUPs is consistently close to zero, which is the value of the population mean. This requires a balance of genotypes with above-average and below-average performance, resulting in maximum diversity. Therefore, there are potential unexplored applications of SEM and SEV as criteria for optimizing the degree of replication, the training set composition (Isidro y Sánchez and Akdemir 2021), maximizing variability in genomic mating (Akdemir and Sánchez 2016; Woolliams and Thompson 1994; Kinghorn 1999), etc.

### Comparison of the different simulation strategies

In this work, we have tested marker-based simulations (Fixed and ESS) and a non-marker-based simulation (MVN). The latter does not require knowledge of the actual values of the variance components, relying instead on REML-estimated scaling factors, such as $\sigma ^2_{a^*} \ne \text {var}({\varvec{a}})$. Conversely, the marker-based strategies need the actual values of the variance components, such as $\sigma ^2_{a} = \text {var}({\varvec{a}})$. As discussed earlier, these values can only be reliably estimated within a reasonable error margin using the PEV methodology described in this work. The MVN simulation has numerous drawbacks, whereas the Fixed and ESS ones are recommended, highlighting the importance of the accurate estimation of variance component estimation through the PEV methodology.

To justify our argument against the MVN simulation, we will review the list of ideal requirements for a simulation outlined in the introduction. We will also use this to compare the ESS with the Fixed strategies.

For simplicity, this work focuses on simulating field trials within a single year. However, for across-year and across-generations simulations, it is important to calculate marker effects that remain consistent across generations. Both marker-based simulations (Fixed and ESS) can do this, but the MVN one can only do it partially. It is possible to obtain additive marker effects from additive values as $\varvec{\beta } = M'(MM')^{-1}{\varvec{a}}$ (Henderson (1977), see Appendix 7 for more details). However, to our knowledge, it is not possible to do the same for the interaction effects, which would limit the MVN simulation across generations.

Furthermore, extracting the marker effects from the additive values in the MVN approach does not provide any control over the desired distribution of the marker effects (i.e., trait architecture), while both Fixed and ESS simulations can control from which distribution the markers are sampled. Moreover, the MVN approach would independently sample additive and interaction effects such as epistasis, which is not realistic because the magnitude of the epistasis between two QTL is likely to be affected by the size of their additive values. In other words, the different effects are not coherent in the MVN simulation. This issue can be resolved in the Fixed and ESS simulations by calculating interaction effects as the product of additive effects weighted by an interaction intensity.

Regarding the control of the variance components, as shown in Fig. 3, environmental, additive and additive by environment were significantly different from the target variance in the MVN simulation. The inclusion of the *G* matrix and the covariance between the environments in the simulation makes the control of variance components challenging in the MVN simulation, as the nonzero covariances distort the overall variation in unpredictable ways. The ESS and Fixed simulations solve this by scaling the effects to the desired target after they are sampled.

Another similar problem of the MVN simulation is its inability to ensure that the *AxL* matrix of genotype by location interactions is double-centered, which would add undesired variation to the additive and location main effects. This is not a problem for the ESS and Fixed simulations, which can adjust the genotype by environment variance after double-centering it.

Finally, both MVN and ESS simulations can consider sampling variance, but the Fixed approach does not. This is the only difference between the ESS and Fixed simulations, but in our opinion, it makes ESS the preferable choice. Including sampling variance allows for heterogeneous spatial and residual variances across locations, heterogeneous location variance across years, etc. All of these occur routinely in empirical field trials. Therefore, including them in simulations is essential for matching reality as much as possible. Furthermore, even when simulating a single field trial within a single year, fixing all variance components to always be exactly equal to the target is not ideal. The genetic variance components estimated from empirical data are not the true values; they are simply our best estimate. Allowing the variance components to fluctuate in different repetitions according to SEV, results in a comprehensive exploration of different combinations of likely variance values centered around the desired target with a theoretically sound distribution.

Furthermore, the ESS simulation is very flexible. While this work used it to simulate some complex interaction terms, it is also suitable for other effects not showcased in this work for simplicity. Examples include dominance with or without heterosis, epistasis, autoregressive spatial trends within each field, interaction between non-additive genetic effects and the location, and even effects not usually considered, such as genotypic by spatial interactions within a field.

In summary, both the Fixed and ESS methodologies offer significant advantages over the MVN simulation, while ESS is superior to the Fixed method in its ability to consider sampling variance. The main challenge in the Fixed and ESS simulations was finding a good estimation for the target variance components from empirical data. This can be done with minimal error with the PEV methodology described in this work, which allows simulations to very closely mimic empirical field trials. This can be invaluable for testing novel methodologies and exploring different breeding schemes under realistic circumstances at a minimal cost.

## Conclusions

This work highlights the limitations in current methodologies for extracting variance components from a LMM fitted on empirical data and proposes a PEV-based alternative to minimize bias. This approach can significantly improve the accuracy of important metrics such as heritability and response from selection, aiding decision-making in breeding programs. Although this study focuses primarily on additive variance for simplicity, the PEV method is applicable to estimating the variance component of any random effect in a LMM.

Accurate estimation of variance components is crucial to ensure that the target variance in simulation studies closely matches the empirical datasets they aim to replicate. Additionally, to improve simulation accuracy, it is important to account for the sampling variance of variance components across simulation runs. To address this, we have introduced a generalized ESS for the sample variance in any multivariate normal distribution, accommodating heteroscedasticity, correlations, and heterogeneous means. This approach ensures that the variance of simulation terms matches their theoretical distribution across repetitions. Consequently, our simulation can reflect the varying variance components observed across generations in empirical breeding programs.

## Supplementary Information

Below is the link to the electronic supplementary material.

Supplementary File 1: Small R script showcasing the difficulty of summarizing in a single scalar the overall variability of a multivariate normal distribution (r 2 KB)

Supplementary File 2: It contains the code needed to run the ESS simulation (r 21 KB)

Supplementary File 3: It contains the code used to validate the proposed generalized SEM and SEV. This code was used to generate the results in Table 2 (r 10 KB)

Supplementary File 4: Additive (*G*) and dominance ($G_d$) relationship matrices computed from the oat dataset (Canales et al. 2021b). These matrices are used as an input for the code in Supplementary File 3 (rdata 7176 KB)

## Data Availability

The oat dataset used in this work is available in (Canales et al. 2021b), and the spruce dataset is available in Beaulieu et al. (2014).

## Appendices

### Appendix 1. VanRaden assumptions

Here, we will show the derivation of the VanRaden (2008) genomic additive relationship matrix (*G*) to explain why it relies on unrealistic assumptions that prevent the REML estimation of $\sigma ^2_{a^*}$ from being meaningful. We will also argue that the overly simplistic assumptions mostly affect the scaling of *G* instead of its covariance structure. As genomic BLUP estimates depend only on the latter, the *G* matrix is valid for performing genomic predictions; however, it is not suitable for variance component estimation.

For a population with a degree of ploidy $\phi$ (e.g., $\phi =2$ for diploid species), let *Z* be the column-centered matrix of marker scores with *n* individuals in rows and *p* SNP marker positions in columns, with $\varvec{\beta }$ being the associated vector of *p* additive marker effects. The variance of the additive values (${\varvec{a}}$) can be expressed as $\text {var}({\varvec{a}}) = \text {var}(Z\varvec{\beta }) = \sigma ^2_a$ and the variance-covariance matrix of the additive values ($\text {cov}({\varvec{a}}) = G\sigma ^2_a$) can be calculated if the following conditions are assumed for any individual *i* and marker positions *j*, *k*:

- Normally distributed, i.i.d. marker effects: $\varvec{\beta } \sim \text {MVN}({\varvec{0}}, I\sigma ^2_\beta )$
- Independent markers (no linkage disequilibrium). Only if this is met: $\text {var}(Z[i,j]\beta _j + Z[i,k]\beta _k) = \text {var}(Z[i,j]\beta _j) + \text {var}(Z[i,k]\beta _k)$. This assumption can be relaxed to $\sum _{j=1}^p \sum _{k \ne j} \text {cov}(Z[i,j]\beta _j, Z[i,k]\beta _k) = 0$
- Marker effects are independent from marker scores. Only if this is true: $\text {var}(Z[i,j]\beta _j) = \text {var}(Z[i,j]) \text {var}(\beta _j)$
- Marker scores in *Z* are assumed to come from a binomial distribution. For a marker position *j* with minor allele frequency $p_j$, the variance of its binomial distribution is $var(Z[1:n,j]) = \phi p_j(1-p_j)$, where *Z*[1 : *n*, *j*] refers a subset of *Z* with all *n* elements from its $j^{th}$ column.

From these assumptions, we can derive that:

$$\begin{aligned} & \sigma ^2_a = \text {var}({\varvec{a}}) = \text {var}(Z\varvec{\beta }) = \text {var}(\varvec{\beta })\sum _{j=1}^p \text {var}(Z[1:n,j]) \\ & \quad = \sigma ^2_\beta \phi \sum _{j=1}^p p_j(1-p_j) \end{aligned}$$

Now, we can solve this equation for $\sigma ^2_\beta$:

10 $$\begin{aligned} \sigma ^2_\beta = \frac{\sigma ^2_a}{\phi \sum _{j=1}^p p_j(1-p_j)} \end{aligned}$$

Next, if we now consider *Z* as a fixed incidence matrix, we can show that:

11 $$\begin{aligned} G\sigma ^2_a = \text {cov}({\varvec{a}}) = \text {cov}(Z\varvec{\beta }) = Z \text {cov}(\varvec{\beta }) Z^T = Z I \sigma ^2_\beta Z^T = ZZ^T\sigma ^2_\beta \end{aligned}$$

Finally, substituting Eq. (10) into Eq. (11), we obtain that:

$$\begin{aligned} & G\sigma ^2_a = ZZ^T\sigma ^2_\beta = \frac{ZZ^T}{\phi \sum _{j=1}^p p_j(1-p_j)}\sigma ^2_a \\ & \quad \rightarrow G = \frac{ZZ^T}{\phi \sum _{j=1}^p p_j(1-p_j)} \end{aligned}$$

Which is the VanRaden relationship matrix. Of all the assumptions needed, we argue that the normally distributed, i.i.d. marker effects can be overly simplistic for certain trait architectures but may also be realistic. In contrast, the binomial distribution of the marker scores is only true if the population is in HWE, which is rare in breeding populations. Next, the absence of linkage disequilibrium is never met in breeding populations even if markers that are not physically linked are considered (see Lehermeier et al. (2017) for an in-depth explanation). Finally, the independence of marker effects from marker scores is questionable, as very favorable alleles are selected in breeding, increasing their frequency and potentially causing a correlation between marker scores and marker effects.

Therefore, while the covariance structure of *G* (given by $ZZ^T$) does not require most of these unrealistic assumptions, the scaling factor $\phi \sum _{j=1}^p p_j(1-p_j)$ does, which makes the *G* matrix unsuitable for variance component estimation via REML. The only assumption needed for the covariance structure $ZZ^T$ is the presence of HWE, which is frequently not met in breeding populations. This issue has been tackled by the additive relationship matrix developed for the NOIA model (Vitezica et al. 2017), although it does not solve the issue of the proper scaling of the matrix. For more details, see Appendix 5.

### Appendix 2. M2 methodology for variance estimation

In this appendix, we will show the derivation of the M2 methodology for estimating the additive variance component described in Lehermeier et al. (2017) and we will justify why it is equivalent to calculating the variance of the vector of genomic BLUPs.

Let *Z* be the column-centered matrix of marker scores with *n* individuals in rows and *p* SNP marker positions in columns. Then, the covariance matrix between markers is $\Sigma _M = \frac{Z^T Z}{n}$. Next, we know that ${\varvec{a}} = Z\varvec{\beta }$, where ${\varvec{a}}$ is a vector of true breeding values and $\varvec{\beta }$ is a vector of true additive marker effects. It is also known that the breeding values are mean-centered ($\bar{a} = 0$). From this:

$$\begin{aligned} \text {var}({\varvec{a}}) =\,& \frac{\sum _{i=1}^n (a_i - \bar{a})^2}{n} = \frac{\sum _{i=1}^n (a_i)^2}{n} \\ =\,& \frac{\sum _{i=1}^n (Z[i,1:p]\varvec{\beta })^2}{n} \end{aligned}$$

Which, in matrix notation, is equivalent to:

$$\begin{aligned} \text {var}({\varvec{a}}) = \frac{\sum _{i=1}^n (Z[i,1:p]\varvec{\beta })^2}{n} = \frac{\varvec{\beta }^T Z^T Z \varvec{\beta }}{n} = \varvec{\beta }^T \Sigma _M \varvec{\beta } \end{aligned}$$

Please note that, in the denominator of the sample variance, we are using *n* instead of $n-1$. This adjustment is because the subtracting one is a correction factor for the degree of freedom expended in the calculation of the mean, which is required for computing variance. However, in this case, we are assuming that the population mean is equal to zero. Therefore, we do not need to calculate it, allowing us to retain all degrees of freedom for variance calculation.

From the derivation above, it is evident that the variance of the genomic BLUPs can be expressed similarly:

$$\begin{aligned} \text {var}(\varvec{{\hat{a}}}) = \varvec{{\hat{\beta }}}^T \Sigma _M \varvec{{\hat{\beta }}} \end{aligned}$$

Therefore, only if the true marker effects $\varvec{\beta }$ are known it is possible to estimate the true population variance as $\sigma ^2_a = \text {var}({\varvec{a}}) = \varvec{\beta }^T \Sigma _M \varvec{\beta }$. However, the true marker effects are never known in empirical datasets, and instead, the BLUP estimates $\varvec{{\hat{\beta }}}$ are available. This only allows us to compute the variance of the BLUPs, which, due to shrinkage, have lower variance than the true additive values. In fact, calculating $\varvec{{\hat{\beta }}}^T \Sigma _M \varvec{{\hat{\beta }}}$ outputs the same value as calculating the variance of the vector of estimated breeding values ($\varvec{{\hat{a}}}$) as $\text {var}(\varvec{{\hat{a}}}) = \frac{\sum _{i=1}^n (\hat{a_i})^2}{n}$, with the latter being more computationally efficient as it does not need to compute the usually extremely large $\Sigma _M$ matrix and can be calculated directly from the predictions of a GBLUP model without the need of estimating marker effects. we have also checked empirically for several traits in several datasets that $\text {var}(\varvec{{\hat{a}}}) = \varvec{{\hat{\beta }}}^T \Sigma _M \varvec{{\hat{\beta }}}$ is exactly equivalent to $\text {var}(\varvec{{\hat{a}}}) = \frac{\sum _{i=1}^n (\hat{a_i})^2}{n}$ and it was true in all scenarios.

In summary, unless there is extremely high replication of the genotypes or extremely high heritability (both of which make shrinkage negligible and $\varvec{\beta } \sim \varvec{{\hat{\beta }}}$), $\varvec{{\hat{\beta }}}^T \Sigma _M \varvec{{\hat{\beta }}}$ will be an underestimation of $\sigma ^2_a$. In Lehermeier et al. (2017), the replication was high (four replicates) and heritability was extremely high (around 0.9), which explains why this methodology performed well for them but poorly in our lower-replication, lower-heritability dataset.

### Appendix 3. Generalized effective sample size derivation

It is well known that the standard errors in the estimation of sample mean (SEM) and sample variance (SEV) are heavily influenced by sample size (*n*). When the available data is an independent, identically distributed (i.i.d.) sample from a normal distribution with variance $\sigma ^2$, the standard errors can be calculated as follows:

$$\begin{aligned} & \text {SEM} = \frac{\sigma }{n^{1/2}}\\ & \text {SEV} = \frac{\sqrt{2}\sigma ^2}{(n-1)^{1/2}} \end{aligned}$$

Here, we will derive how SEM and SEV and their associated effective sample sizes ($\text {ESS}_\mu$ for the mean and $\text {ESS}_{\sigma ^2}$ for the variance) can be calculated for any multivariate normal distribution.

#### Initial definitions

We commence by defining a vector of correlated random variables ${\varvec{X}} = \{X_1, X_2,\ldots , X_n\}$, adhering to a multivariate normal distribution, ${\varvec{X}} \sim \text {MVN}(\varvec{\mu }, \Sigma )$. It is imperative to note the variance of a sum of normally distributed random variables is the summation of their variances and twice their pairwise covariances:

12 $$\begin{aligned} \text {var}\bigg (\sum _{i=1}^n X_i\bigg ) =\,& \sum _{i=1}^n \text {var}(X_i) + 2\sum _{i=1}^n \sum _{j \ne i} \text {cov}(X_i,X_j) \nonumber \\ =\,& \varvec{1^T}\Sigma {\varvec{1}} \end{aligned}$$

where ${\varvec{1}}$ is a vector of ones. Therefore, the variance of the sum of all $X_i$ is just the sum of all elements of their variance-covariance matrix $\Sigma$. This property will be used extensively in the subsequent sections.

#### Effective sample size for the sample mean

Although SEM is not directly of interest in this work, it is a prerequisite for calculating SEV. Using Eq. (12), we can easily obtain a way to calculate SEM for correlated and heteroscedastic samples, which can be subsequently used to obtain an ESS. We will call this the generalized standard error of the mean ($\text {SEM}_g$)

13 $$\begin{aligned} \text {SEM}_g^2 &= \text {var}(E(X)) = \text {Var}\left( \frac{\sum _{i=1}^n X_i}{n}\right) = \frac{\text {var}(\sum _{i=1}^n X_i)}{n^2} \nonumber \\ & = \frac{\varvec{1^T}\Sigma {\varvec{1}}}{n^2} \end{aligned}$$

To proceed to the calculation of $\text {ESS}_\mu$, we first need to provide a clear definition of it. $\text {ESS}_\mu$ is the number of i.i.d. observations that contain the same amount of information for the calculation of the mean as the correlated, heteroscedastic samples of interest. Therefore, the equation of the $\text {SEM}_g$ (Eq. 13) will be equivalent to the classical equation for the SEM (Eq. 6, assumes i.i.d. observations) if the sample size *n* is replaced by $\text {ESS}_\mu$ in the latter. Thus, $\text {ESS}_\mu$ acts as a correction factor that compensates for the correlated and heteroscedastic observations. When the observations are heteroscedastic, the value for the variance of the distribution required in Eq. (6) can be computed as the average variance in $\Sigma$, which is calculated as $\bar{\sigma ^2} = \text {tr}(\Sigma )/n$, where $\text {tr}()$ is the trace of a matrix. This average is equivalent to the average of the eigenvalues since the trace of a covariance matrix is the sum of its eigenvalues.

14 $$\begin{aligned} & \text {SEM}_g^2 = \frac{\varvec{1^T}\Sigma {\varvec{1}}}{n^2} = \frac{\bar{\sigma ^2}}{\text {ESS}_\mu }; \; \text {ESS}_\mu = \frac{\bar{\sigma ^2} n^2}{\varvec{1^T}\Sigma {\varvec{1}}} = \frac{\text {tr}(\Sigma )}{\varvec{1^T}\Sigma {\varvec{1}}} n \end{aligned}$$

This is similar to the equation for the calculation of $\text {ESS}_\mu$ proposed in Griffith (2005) and Griffith (2008) in the context of spatial statistics and autoregression (although in the former the inverse of $\Sigma$ is required instead of using $\Sigma$ directly). Here, we have generalized it, showing that it holds for any multivariate normal distribution.

#### Effective sample size for the sample variance

The ESS for the variance ($\text {ESS}_{\sigma ^2}$) has a different value than the one for the mean ($\text {ESS}_{\mu }$), but it can be computed using a similar concept. First, we will need a way to calculate the generalized standard error of the variance for correlated, heteroscedastic samples with heterogeneous means ($\text {SEV}_g$):

15 $$\begin{aligned} \text {SEV}_g^2 =\,& \text {var}\bigg (s^2\bigg ) = \text {Var} \left( \frac{\sum _{i=1}^n [(X_i - \bar{X})^2]}{n-1} \right) \nonumber \\ =\,& \frac{\text {var}(\sum _{i=1}^n [(X_i - \bar{X})^2])}{(n-1)^2} \end{aligned}$$

In Eq. (15), we know that $X_i \in {\varvec{X}} \sim \text {MVN}(\varvec{\mu }, \Sigma )$. We also know that $\bar{X}$ is the sample mean, which follows a univariate normal distribution $\bar{X} \sim N(\bar{\mu }, \text {SEM}^2)$, where $\text {SEM}^2$ can be easily computed as shown in Eq. (13) and $\bar{\mu }$ is the average of the vector $\varvec{\mu }$, i.e., $\bar{\mu } = n^{-1}\sum _{i=1}^n \mu _i$

Before proceeding, we need to find the distribution of $(X_i - \bar{X})^2$. First, $X_i - \bar{X}$ is the result of subtracting two normally distributed variables, which will follow a normal distribution: $(X_i - \bar{X}) \sim \text {MVN}(\varvec{\mu }-\bar{\mu }, \Sigma _{1})$. Finding $\Sigma _{1}$ will be the initial step, which can be done from $\Sigma _{1}[i,j] = \text {cov}[(X_i-\bar{X}), (X_j-\bar{X})]$, where [*i*, *j*] refers to the element in the $i{\text {th}}$ row and $j{\text {th}}$ column of the matrix. Using the fact that the covariance is a bilinear operator:

16 $$\begin{aligned} & \text {cov}[(X_i-\bar{X}), (X_j-\bar{X})] = \text {cov}(X_i,X_j) + \text {cov}(-\bar{X},-\bar{X})\nonumber \\ & \quad + \text {cov}(X_i,-\bar{X}) + \text {cov}(X_j,-\bar{X}) \end{aligned}$$

where $\text {cov}(X_i,X_j) = \Sigma [i,j]$, $\text {cov}(-\bar{X},-\bar{X}) = \text {var}(\bar{X}) = \text {SEM}^2$ and $\text {cov}(X_i,-\bar{X})$ can be computed as follows:

17 $$\begin{aligned} \text {cov}(X_i, -\bar{X}) =\,& -\text {cov}\bigg(X_i, \frac{1}{n} \sum _{j=1}^n X_j\bigg) \nonumber \\ =\,& -\frac{1}{n} \sum _{j=1}^n [\text {cov}(X_i, X_j)] = -\frac{\Sigma [i,1:n]{\varvec{1}}}{n} \end{aligned}$$

where [*i*, 1 : *n*] for the matrix $\Sigma$ indicates that a subset comprising all elements within the $i{\text {th}}$ row vector is taken. That way, from Eq. (17) we can see that the covariance between an observation $X_i$ and its sample mean is simply the average of the $i{\text {th}}$ row of $\Sigma$.

In summary, from the results of Eqs. (16) and (17), we can build the $\Sigma _1$ matrix as follows for all $i,j \in \{1,2,\ldots , n\}$:

18 $$\begin{aligned} \Sigma _1[i,j] = \Sigma [i,j] + \text {SEM}^2 - \frac{\Sigma [i,1:n]{\varvec{1}}}{n} -\frac{\Sigma [j,1:n]{\varvec{1}}}{n} \end{aligned}$$

Finally, $\Sigma _1$ is the variance-covariance matrix of $(X_i - \bar{X})$, but we need its square. Therefore, we can define $\Sigma _2 = \text {cov}[(X_i - \bar{X})^2]$. The equations to calculate the variance-covariance of the square of a multivariate normal distribution are known, which allows us to easily compute $\Sigma _2$ from $(X_i - \bar{X})^2 \sim [\text {MVN}(\varvec{\mu }-\bar{\mu }, \Sigma _{1})]^2$. For all $i,j \in \{1,2,\ldots , n\}$:

19 $$\begin{aligned} \Sigma _{2}[i,j] = 2(\Sigma _1[i,j])^2 + 4\Sigma _1[i,j] (\mu _i-\bar{\mu })(\mu _j-\bar{\mu }) \end{aligned}$$

Now that $\text {cov}[(X_i - \bar{X})^2] = \Sigma _2$ is known, we can calculate $\text {var}(\sum _{i=1}^n [(X_i - \bar{X})^2])$ in Eq. (15) as the sum of all elements of $\Sigma _2$ (as shown in Eq. (12)). Therefore, continuing from Eq. (15), we can calculate $\text {SEV}_g^2$ as follows:

20 $$\begin{aligned} \text {SEV}_g^2 = \frac{\text {var}(\sum _{i=1}^n [(X_i - \bar{X})^2])}{(n-1)^2} = \frac{\varvec{1^T}\Sigma _2{\varvec{1}}}{(n-1)^2} \end{aligned}$$

Similarly to $\text {ESS}_{\mu }$, we can define $\text {ESS}_{\sigma ^2}$ as the number of i.i.d. observations with homogeneous means that contain the same amount of information for the calculation of the sample variance as the heteroscedastic, correlated samples with heterogeneous means of interest. Therefore, the equation of the $\text {SEV}_g$ (Eq. 20) will be equivalent to the classical equation for the $\text {SEV}$ (Eq. 6, assumes i.i.d. observations and homogeneous means) if the sample size *n* is replaced by $\text {ESS}_{\sigma ^2}$ in the latter. Thus, $\text {ESS}_{\sigma ^2}$ acts as a correction factor that compensates for the correlated, heteroscedastic observations with heterogeneous means. As previously explained for the $\text {SEM}$, in the presence of heteroscedasticity, the value for the variance of the distribution required in Eq. (6) can be computed as the average variance in $\Sigma$, calculated as $\bar{\sigma ^2} = \text {tr}(\Sigma )/n$.

$$\begin{aligned} \text {SEV}_g^2 = \frac{\varvec{1^T}\Sigma _2{\varvec{1}}}{(n-1)^2} = \frac{2(\bar{\sigma ^2})^2}{\text {ESS}_{\sigma ^2}-1}; \; \text {ESS}_{\sigma ^2} = \frac{2(\bar{\sigma ^2})^2(n-1)^2}{\varvec{1^T}\Sigma _2{\varvec{1}}} + 1 \end{aligned}$$

The main advantage of calculating ESS over computing the *SEM* and *SEV* directly from Eqs. (13) and (20) respectively, is that ESS allows summarizing in a single, intuitive scalar how efficient our sample is, which could be used to characterize the distribution and to optimize the sampling method to minimize the information loss (or maximize gain) due to correlations, heteroscedasticity, and heterogeneous means. Furthermore, while SEM and SEV are sensitive to the multiplication of the ${\varvec{X}}$ vector by a scalar, both $ESS_\mu$ and $\text {ESS}_{\sigma ^2}$ are insensitive to it, which make them a more consistent metric.

### Appendix 4. Confidence interval for unbiased variances in simulation

Here, we will show how we built the confidence interval in Fig. 3. We will exemplify it using the additive effects, but a similar methodology was employed for all terms in the simulation.

Additive effects are assumed to follow a normal distribution, with variance equal to the PEV estimation of $\sigma ^2_a$ and variance-covariance structure equal to GN. The 500 simulation iterations can be viewed as 500 independent samples of the additive values, and the variance of each sample will be distributed following a gamma distribution of mean equal to the PEV estimate of $\sigma ^2_a$ and standard deviation equal to SEV. SEV can be calculated as in Eq. (7) using $\Sigma = \text {GN}\sigma ^2_a$, which would yield $\bar{\sigma ^2} = \frac{\text {tr}(\Sigma )}{n} = \sigma ^2_a$.

As a result, we know the theoretical distribution for the additive variance in a simulation run: $s^2_a \sim \Gamma (\mu = \sigma ^2_a, \sigma = \text {SEV})$. As the 500 simulation runs are independent, following the central limit theorem, the mean of their variances is normally distributed: $E(s^2_a) \sim N(\mu = \sigma ^2_a, \sigma = \text {SEM}_{s^2_a})$. Here, $\text {SEM}_{s^2_a}$ refers to the standard error of the mean for the 500 $s^2_a$ values, which can be calculated as $\text {SEM}_{s^2_a} = \frac{\text {SEV}}{\sqrt{500}}$ thanks to the fact that the 500 simulation runs are i.i.d. Therefore, the average value of $s^2_a$ across the 500 simulation runs ($\bar{s^2_a}$) will be within $\sigma ^2_a \pm 2\text {SEM}_{s^2_a}$ with a probability of approximately 0.95. Thus, the ‘standard error units‘ used in the horizontal axis of Fig. 3 correspond to the deviation of $\bar{s^2_a}$ from $\sigma ^2_a$ measured in units of $\text {SEM}_{s^2_a}$.

### Appendix 5. Additive variance estimation with the NOIA relationship matrix

The NOIA model makes use of an additive relationship matrix ($G_{\text {NOIA}}$) that can be considered a generalization of the VanRaden matrix that does not require the assumption that the population is in HWE (Vitezica et al. 2017). Briefly, to calculate it, an incidence matrix *H* with individuals in rows and marker positions in columns has to be created. In diploid crops, for individual *i* and marker *j*, it has the following value:

21 $$\begin{array}{*{20}l} {H[i,j] = - ( - p_{{Aa}} - 2p_{{aa}} )} \hfill & {{\text{for}}} \hfill & {{\text{genotype}}} \hfill & {AA} \hfill \\ {H[i,j] = - (1 - p_{{Aa}} - 2p_{{aa}} )} \hfill & {{\text{for}}} \hfill & {{\text{genotype}}} \hfill & {Aa} \hfill \\ {H[i,j] = - (2 - p_{{Aa}} - 2p_{{aa}} )} \hfill & {{\text{for}}} \hfill & {{\text{genotype}}} \hfill & {Aa} \hfill \\ \end{array}$$

where *A* and *a* are the two possible alleles in locus *j*, $p_{Aa}$ is the frequency of the heterozygous genotype in the population and $p_{aa}$ is the frequency of the genotype homozygous for *a* in the population. The *H* matrix is equivalent to the *Z* matrix in Eq. (1) when the population is under HWE (Vitezica et al. 2017). Next, from the *H* matrix, we can calculate $G_{\text {NOIA}}$ as follows:

22 $$\begin{aligned} G_{\text {NOIA}} = \frac{HH^T}{tr(HH^T)/n} \end{aligned}$$

It is important to note that $G_{\text {NOIA}}$ is scaled similarly to GN, dividing it by the mean of its diagonal values.

Next, we fitted model 5 using $G_{\text {NOIA}}$ instead of *G* as the additive relationship matrix, i.e., ${\varvec{a}} \sim \text {MVN}({\varvec{0}}, G_{\text {NOIA}}\sigma ^2_{a^*})$ and calculated the additive variance component $\sigma ^2_a$ in three ways: unbiased estimate ($\sigma ^2_a = \frac{\sum _{i=1}^n ({\hat{g}}_i - \bar{{\hat{g}}})^2}{n-1} - \sigma ^2_\epsilon$), REML estimate ( $\sigma ^2_a = \sigma ^2_{a^*}$) and PEV estimate ( $\sigma ^2_a = \frac{\sum _{i=1}^n {\hat{a}}_i^2}{n} + \frac{\text {tr}(\text {PEV})}{n}$).

Firstly, as the covariance structure in $G_{\text {NOIA}}$ is different to that in *G* and *GN*, it is possible for the unbiased estimate of the additive variance to be different in the model fitted with $G_{\text {NOIA}}$ and the model relying on *G*. As seen in Table 3, these differences were negligible in the balanced datasets regardless of whether or not they were in HWE. However, for the unbalanced oat dataset, there were substantial differences in additive variance.

**Table 3:** Comparison of the unbiased variance component estimation when using the *G* matrix or the $G_{\text {NOIA}}$ matrix for fitting the model

Dataset	Experimental design	Trait	${\sigma ^2_{\text {Unbiased}}}$, *G* matrix	${\sigma ^2_{\text {Unbiased}}}$, ${G_{\text {NOIA}}}$ matrix
Oat	Balanced	Yield	0.242	0.243
Oat	Balanced	Biomass	0.983	0.990
Oat	Balanced	Height	136.71	135.68
Oat	Balanced	Heading days	87.27	87.33
Oat	Unbalanced	Yield	0.299	0.327
Oat	Unbalanced	Biomass	1.100	1.334
Oat	Unbalanced	Height	147.38	132.12
Oat	Unbalanced	Heading days	85.48	90.68
Spruce	Balanced	Diameter	0.165	0.165
Spruce	Balanced	Height	0.227	0.227
Spruce	Balanced	Density	0.356	0.356

For the unbalanced datasets, the average variance across 20 repetitions of randomly dropping observations from the full dataset are presented

Finally, within the model fitted with the $G_{\text {NOIA}}$ matrix, Table 4 contains the result of comparing the unbiased estimation of additive variance with the REML and PEV estimates. It is important to note that the variance values in Tables 3 and 4 are different because the former are the raw estimates and the latter have been standardized. In Table 4, it can be seen that in all cases the bias of the PEV estimate was minimal, showcasing its robustness. In contrast, the REML estimate tended to be very poor in the oat dataset, specially for the unbalanced design. In summary, the PEV method is preferable to REML regardless of the relationship matrix used in the model.

**Table 4:** Comparison of the variance component estimation between the different methods in the oat and spruce datasets

Dataset	Experimental sesign	Trait	$\sigma ^2_{\text {Unbiased}}$	$\sigma ^2_{\text {NOIA}}$	$\sigma ^2_{\text {PEV}}$
Oat	Balanced	Yield	1	0.947	0.995
Oat	Balanced	Biomass	1	1.313	0.995
Oat	Balanced	Height	1	1.742	0.997
Oat	Balanced	Heading days	1	0.970	0.999
Oat	Unbalanced	Yield	1	2.671	0.990
Oat	Unbalanced	Biomass	1	2.901	0.987
Oat	Unbalanced	Height	1	2.585	0.984
Oat	Unbalanced	Heading days	1	2.303	0.999
Spruce	Balanced	Diameter	1	1.006	0.993
Spruce	Balanced	Height	1	0.948	0.998
Spruce	Balanced	Density	1	0.936	0.998

All estimates have been extracted from a model fitted with the $G_{\text {NOIA}}$ matrix as covariance structure of the additive effect. For the unbalanced datasets, the average variance across 20 repetitions of randomly dropping observations from the full dataset are presented. The column for $\sigma ^2_{\text {Unbiased}}$ displays the difference between the variance of the response variable and residual variance. $\sigma ^2_{\text {NOIA}}$ contains the REML estimation of the variances. $\sigma ^2_{\text {PEV}}$ estimates the variance component with the PEV method. For ease of interpretation, the variance estimates in each row have been standardized in such a way that the value of $\sigma ^2_{\text {Unbiased}}$ is equal to one

### Appendix 6. Variance estimation for independent, identically distributed effects

To test the PEV method of variance estimation on i.i.d. genotypic values, the following model was used for the oat dataset:

$$\begin{aligned} {\varvec{y}} = X_{L} {\varvec{L}} + X_{Block} \varvec{Block} + Z_g{\varvec{g}} + \varvec{\epsilon } \end{aligned}$$

where ${\varvec{y}}$ is a vector of phenotypes, ${\varvec{L}}$ is a vector of fixed location effects, $\varvec{Block}$ is a vector of fixed block effects nested within location, ${\varvec{g}} \sim MVN({\varvec{0}}, I\sigma ^2_g)$ is a vector of of i.i.d. genotypic effects and $\varvec{\epsilon } \sim \text {MVN}({\varvec{0}}, R)$ is a vector of i.i.d. residuals within each location with heterogeneous variance across locations. $X_{L}$, $X_{Block}$ and $Z_g$ are the corresponding design matrices. The ${\varvec{L}}$ and $\varvec{Block}$ effects were set as fixed because they did not present enough levels for a good variance component estimation by REML. This model was only used in conjunction with the oat dataset as the spruce one was already comprised of adjusted phenotypes without location or block effects.

In this model, the REML estimation for $\sigma ^2_g$ will be unbiased, as the assumed covariance structure is the identity instead of the VanRaden *G* matrix or GN, thus avoiding any scaling issues with *G* and GN. However, the PEV method may present some bias due to the error in extracting the mean prediction error variance from the multivariate PEV matrix. This comparison allows to investigate the magnitude of the bias.

**Table 5:** Evaluation of the PEV methodology for variance estimation for i.i.d. genotypic effects in balanced and unbalanced designs in all traits of the oat dataset

Dataset	Experimental design	Trait	$\sigma ^2_{\text {Unbiased}}$	$\sigma ^2_{\text {PEV}}$
Oat	Balanced	Yield	1.000	1.000
Oat	Balanced	Biomass	1.000	1.001
Oat	Balanced	Height	1.000	1.001
Oat	Balanced	Heading days	1.000	1.001
Oat	Unbalanced	Yield	1.000	1.065
Oat	Unbalanced	Biomass	1.000	1.006
Oat	Unbalanced	Height	1.000	1.071
Oat	Unbalanced	Heading days	1.000	0.999

For the unbalanced dataset, the average values across 20 repetitions of randomly dropping observations from the full dataset are presented. The column for $\sigma ^2_{\text {Unbiased}}$ contains the REML estimation for the variance of the genotypic effects and $\sigma ^2_{\text {PEV}}$ contains the variance component estimate with the PEV method. For ease of interpretation, the variance estimates in each row have been standardized in such a way that the value of $\sigma ^2_{\text {Unbiased}}$ is equal to one

In light of the results in Table 5, we can conclude that the PEV method can be used for i.i.d. effects, as its bias is small, especially for balanced datasets. However, there is no reason to use the PEV method for this kind of effect, as the REML estimate is easy to obtain and will be unbiased for them. The usefulness of this comparison lies in assessing the robustness of the PEV method.

### Appendix 7. Marker effects from breeding values

Here, we will show that the additive marker effects and breeding values contain the same information, and one can be calculated from the other simply using the marker matrix. It is known that breeding values can be computed from marker effects as ${\varvec{a}} = M\varvec{\beta }$, where *M* is the marker matrix, ${\varvec{a}}$ is the vector of additive breeding values and $\varvec{\beta }$ is a vector of additive marker effects. It is less widespread that the inverse is also possible, using the following equation: $\varvec{\beta } = M'(MM')^{-1}{\varvec{a}}$ (Henderson 1977). We can easily prove that this equation holds. To that end, we will start with an equation we know is true:

$$\begin{aligned} {\varvec{a}} = M\varvec{\beta } \end{aligned}$$

If we substitute $\varvec{\beta }$ by $M'(MM')^{-1}{\varvec{a}}$, we get:

$$\begin{aligned} {\varvec{a}} = MM'(MM')^{-1}{\varvec{a}}; \; {\varvec{a}} = {\varvec{a}} \end{aligned}$$

Which proves that the equation $\varvec{\beta } = M'(MM')^{-1}{\varvec{a}}$ holds.

## Declarations

### Statistical software

All analyses were performed in R, version 4.2.2 (2021) with the addition of a Fortran 90 function for the calculation of ESS for increased computational efficiency. Modeling was performed using the package Sommer, version 4.3.1 (Covarrubias-Pazaran 2016), and sampling from multivariate normal distributions relied on the package MASS, version 7.3$-$58.1 (Venables and Ripley 2002).
